# The impact of rural outreach programs on medical students’ future rural intentions and working locations: a systematic review

**DOI:** 10.1186/s12909-018-1287-y

**Published:** 2018-08-14

**Authors:** George E. Johnson, Fredrick Clive Wright, Kirsty Foster

**Affiliations:** 1 Sydney Medical School, University of Sydney, Sydney, NSW 2050 Australia; 2 Concord Clinical School, University of Sydney, Sydney, Australia; 3 Centre for Education and Research on Ageing, Sydney, NSW 2139 Australia; 4 Sydney Medical School, Northern & Kolling Institute, University of Sydney, Sydney, NSW Australia

**Keywords:** Rural placement programs, Employment outcomes, Systematic review, Medical education, Medical students, Rural intentions

## Abstract

**Background:**

Significant investment has been undertaken by many countries into ‘Rural Clinical Training Placement Schemes’ for medical students in order to deal with shortages of trained health care professionals in rural and remote locations. This systematic review examines the evidence base of rural educational programs within medical education and focusses on workforce intentions and employment outcomes. The study provides a detailed description of the methodological characteristics of the literature, thematic workforce outcomes and key related factors are identified, study quality is assessed, and the findings are compared within an international context.

**Methods:**

A systematic review looking at international literature of rural placement programs within medical education between January 2005 to January 2017 from databases including; Medline, Embase, NursingOVID, PubMed and Cochrane. The study adopted the PRISMA protocol. A quality assessment of the literature was conducted based on the Health Gains Notation Framework.

**Results:**

Sixty two papers met the inclusion criteria. The review identified three program classifications; Rural Clinical Placement Programs, Rural Clinical Placement Programs combined with a rural health educational curriculum component and Rural Clinical School Programs. The studies included were from Australia, United States, Canada, New Zealand, Thailand and Africa.

Questionnaires and tracking or medical registry databases were the most commonly reported research tools and the majority were volunteer programs. Most studies identified potential rural predictors/confounders, however a number did not apply control groups and most programs were based on a single site. There was a clear discrepancy in the ideal rural clinical placement length. Outcomes themes were identified related to rural workforce outcomes. Most studies reported that an organised, well-funded, rural placement or rural clinical school program produced positive associations with increased rural intentions and actual graduate rural employment.

**Conclusions:**

Future research should focus on large scale methodologically rigorous multi-site rural program studies, with longitudinal follow up of graduates working locations. Studies should apply pre-and post-intervention surveys to measure change in attitudes and control for predictive confounders, control groups should be applied; and in-depth qualitative research should be considered to explore the specific factors of programs that are associated with encouraging rural employment.

**Electronic supplementary material:**

The online version of this article (10.1186/s12909-018-1287-y) contains supplementary material, which is available to authorized users.

## Background

Rural populations generally have poorer health outcomes, higher mortality rates, lower life expectancy, increased hospitalisation rates, greater chronic disease and higher cancer rates [1, 2]. Factors attributed to these health outcomes include; lifestyle, behavioral factors and lower socioeconomic status. However, another key factor is the lack of effective health services in rural locations and the difficulty in attracting a skilled health workforce to work rurally [1, 3–5].

In the provision of medical services, approximately one half of the world’s population lives in rural areas but these areas are served by only 25% of the total physician workforce [6]. In the United States (US), 20% of the population lives in rural locations; however only 10% of physicians practice in these same locations [7]. In Australia, it is reported that there are 58 practitioners per 100,000 people in remote Australia compared to 196 per 100,000 in metropolitan areas [8]. These workforce issues are of international concern.

Commonly reported barriers in encouraging health professionals to work rurally are: reduced access to continuing medical education, limited professional interaction with peers, heavy responsibilities and workload, substandard medical equipment and facilities, inadequate financial remuneration, social isolation, poor social services, a lack of job opportunities for partners, and inadequate educational opportunities for children [9–11]. Reported factors that encourage interest in rural employment are: a welcoming community, partner employment, family located in a rural area, and the outdoor lifestyle [12]. Furthermore, it is generally accepted that health professionals who grew up in a rural area are more likely to practice in rural locations [13–17].

Attempts to increase the rural health workforce have included; recruitment of qualified doctors from overseas [18, 19], workplace reform [18], improved rural working conditions [18], loan repayment schemes [5, 20], and financial incentives [18, 21]. However, these strategies have achieved mixed results, and over the last two decades there has been a focus on clinical training and education in rural areas, to encourage health professionals to work permanently in rural locations [22–24]. For example, in 1997 the Australian government funded an educational initiative, called the ‘Rural Undergraduate Support and Co-ordination Program (RUSC)‘ [25]; designed to improve curriculum design, rural placements and rural teachers. Furthermore, in 2008, the Australian Federal government committed a $1.1 billion investment in the Health Workforce, which included $500 million in Commonwealth funding for undergraduate clinical training and the establishment of Rural Clinical Schools (RCS) [26].

Ranmuthugala et al. [27] reviewed the evidence of rural exposure on rural medical practice and found that the evidence is inconclusive, as the aspects of rural exposure that are driving positive attitudes toward rural practice are not being identified. They stated that longitudinal analysis is required of these government initiatives aimed at driving workforce re-distribution, and there should be a focus on the structure of these programs in terms of the aspects and factors that impact on rural workforce. A 2017 systematic review focussed on rural training programs in the US and supported the need to further explore the factors of the programs that are contributing to rural practice, with the information being critical for informing strategists and planners. The study stated that currently programs are showing promise but require further refinement and the specific aspects of the training experience that are leading to the program’s success, are poorly understood and need further research [28].

This systematic review considers the current international evidence within medical education and focusses on the how these programs are being conducted and evaluated, and the value of rural clinical placement schemes; specifically, if these programs encourage graduates’ intentions to work rurally and/or lead to actual rural employment. It is hoped by reviewing the methodological characteristics of the programs, the workforce outcomes and related factors, that this will explore the program designs and aspects that are producing positive workforce outcomes.

## Methods

This study adopted the quality appraisal tool and guideline for systematic reviews; ‘Preferred Reporting Items for Systematic reviews and Meta-Analysis’ (PRISMA) protocol instructions, as a guide in the development of the reviews methodology [29, 30].

### Search strategy

**Fig. 1:**
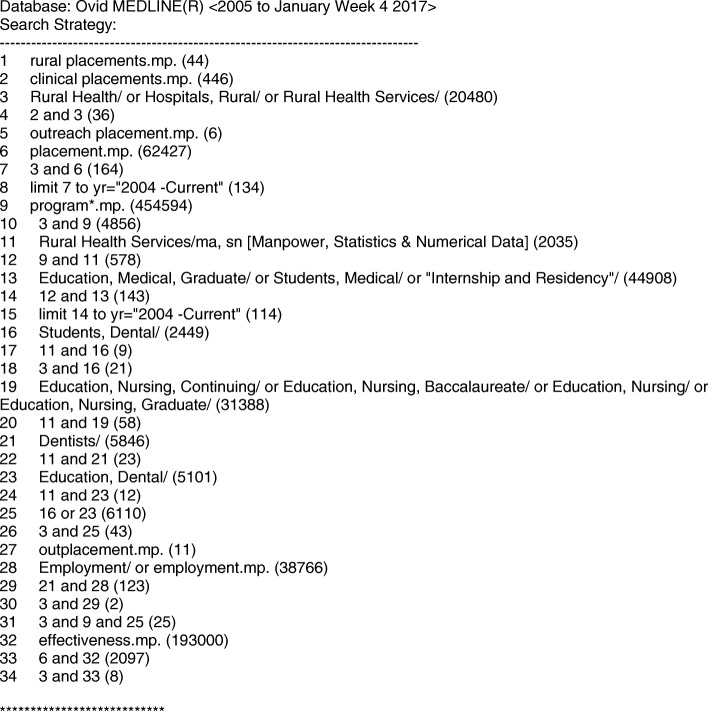
The Search Strategy applied in Ovid MEDLINE (example of search strategy)

The databases searched were Medline, Embase, NursingOVID, PubMed and Cochrane. As PubMed and Cochrane are not OVID databases, the same terms were used as outlined in the example of the search strategy shown in Fig. 1. The Search Strategy provided was applied, with slight variations to fit within the structure of the OVID databases (Embase, Medline, NursingOVID).

The following key words and combinations were used in the search strategy: rural placements, clinical placements, rural health, rural school, rural hospitals, rural health services, rural, outreach, rural health program, rural initiative, education, medical, graduate, students, internship and residency, dental health, dental program, rural doctor, medical education, rural students, rural dentists, rural intentions, rural internships, rural clinical internships, workforce outcomes, working locations, student intentions, nursing, continuing education, longitudinal, nursing graduate/rural, outplacement, employment, dental/medical graduates/employment, clinical clerkship, placement effectiveness, intervention effectiveness, rural strategies and rural employment. This paper presents the findings related to ‘Medical Education Studies’.

All five databases were searched for the period January 2005 to January 2017 (inclusive). Articles available in the databases up to the search date, were included according to the inclusion criteria as stated in Table 1. The search strategy criteria was developed by author one (GJ) who then presented and discussed with the other authors and made modifications as developed through a consultation process (CFW & KF).

**Table 1:** Inclusion and exclusion criteria

Characteristics	Inclusion criteria	Exclusion criteria
Intervention	Considered Medical Education studies involving medical undergraduates. Looked at the evidence of rural clinical placements or rural training in general, in encouraging health professionals to work rurally AND/OR lead to rural employment. Considered programs set in undergraduate or post graduate training and post-graduate tracking programs looking at/related to student rural training programs. Considered other strategies/interventions that encourage health professionals to consider working in rural locations AND/OR lead to rural employment. Focussed on looking at programs/interventions occurring/ involving medical education.	Studies not focused on strategies/interventions measuring either rural intentions or actual rural employment. (Example, studies that are focused on clinical competence of rural clinical placements were not included unless they related the findings to outcomes related to workforce intentions or actual rural employment). Reviews, commentaries, editorials, news and policy briefs were excluded from the results section, however these papers were considered and discussed in the introduction and discussion of this paper. We did not include programs that were not involved/related to medical education programs i.e. rural initiatives aimed at qualified working professionals unrelated to assessing or considering a current or previous educational initiative.
Study design	Randomised control trials, other controlled trials, descriptive and comparative studies.	Systematic reviews, meta-analyses Narrative reviews, editorials, letters, articles. Articles in abstract form only.
Publication	Articles between January 2005 and January 2017 (inclusive)	Studies published prior to 2005
Language	(English language articles)	(Non-English publications)

Adapted from D Yevlahova,* J Satur* Models for individual oral health promotion and their effectiveness: a systematic review

**Fig. 2:**
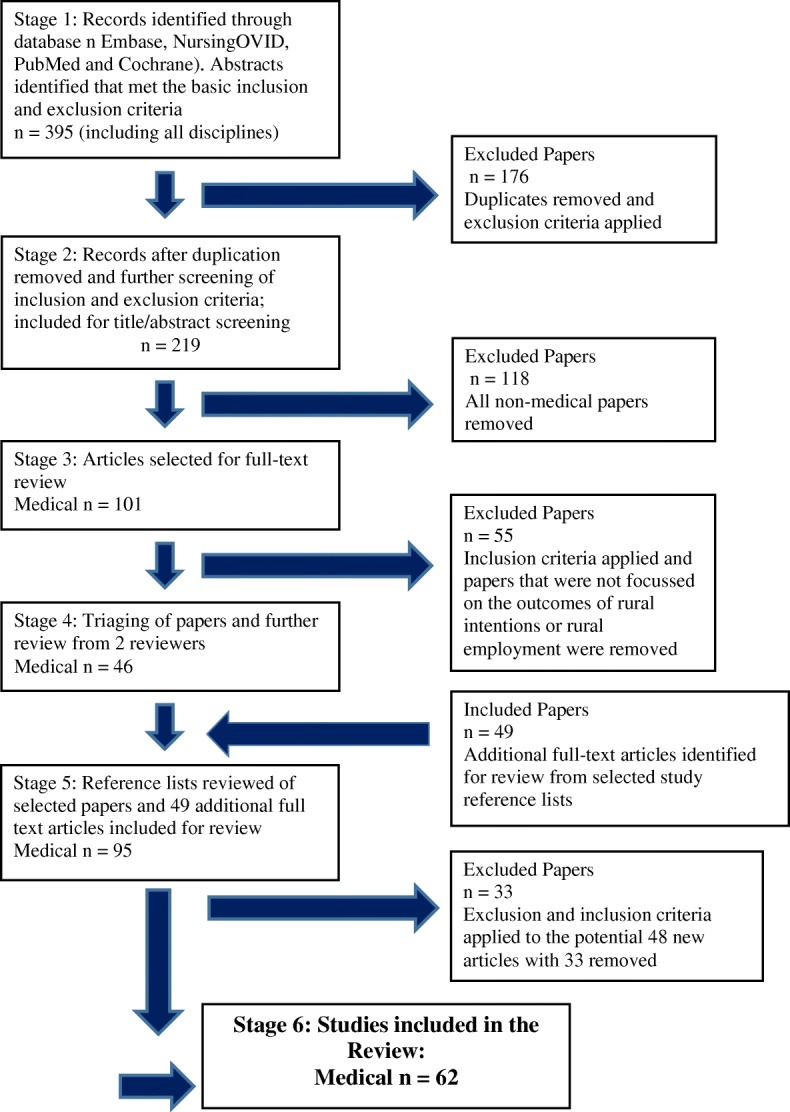
Systematic review flowchart of the literature search and selection process

Figure 2 shows the systematic flowchart of the paper selection process. Stage one involved one author (GJ) independently assessing the titles and abstracts of all the identified articles, to see if they met the basic inclusion and exclusion criteria. Initially all papers related to rural placements or rural clinical initiatives were considered. This resulted in 395 mixed discipline papers for consideration. All articles selected in this review were in English.

At stage two, a reviewer (GJ) applied the exclusion criteria and removed duplicates and irrelevant articles. The two key inclusion criteria were that papers had to consider rural intentions, and rural interest and/or employment outcomes to be considered for further review. In addition, review articles, editorials, or commentaries were removed. We removed reviews as we wanted to assess individual studies/programs and primary evidence within the selected literature, and we planned to compare relevant review evidence within the discussion. At stage three, all non-medical education papers were removed, giving 101 medical education papers.

The 101 articles were then reviewed in full text, and these studies were double screened to ensure consistency, and revisions made to definitions and criteria accordingly. Studies were reviewed based on study characteristics, sampling and recruitment, theoretical framework, methods and results. All studies were coded by one reviewer (GJ) as either a primary, secondary or tertiary paper by their priority of relevance to the inclusion criteria. This was a simple categorisation priority process to assist in the screening and organisation of the papers. A second reviewer (FCW) then independently examined the papers. Discrepancies were discussed until a consensus was reached. (GJ, FCW). The third author (KF) offered advice if an agreement could not be reached between the two authors (GJ & FCW).

From this process, 46 papers were selected. The reference lists of all publications were searched and appropriate papers were included in the final review. Any papers that were added from the reference list review also had their reference list searched and again appropriate papers were included (GJ). This resulted in 62 papers included in this systematic review. Additional file 1: Appendix S1 provides a detailed summary of the 62 selected studies.

### Data extraction and analysis

The review adopted Pharweller et al. [31] extraction process and analysed information related to the studies methodological characteristics, intervention information and reported outcomes from the articles. Two data extraction sheets were developed (GJ) and pilot tested on three articles. The first sheet collected information on the methodological characteristics and adopted the characteristics applied by Pharweller et al. [31] with modification to be specific for this research area. The second sheet collected extracted outcome themes related to workforce outcomes and followed a thematic content analysis approach of categorising trends in the papers findings.

One author (GJ) applied these extraction data sheets and reviewed all the full articles. A second author (FCW) completed an independent review of six random studies to ensure reliability of the data extraction spreadsheets. To ensure quality control the first reviewer (GJ) completed multiple checks of the quantitative summaries against the literature. Authors two (FCW) and three (KF) also reviewed the completed extraction analysis data sheets and discussions occurred during a final consultative review of the extracted information.

### Quality screening process

The review adopted the Quality Assessment process applied by Yevlahova and Satur [32], as it assesses quality for public health and health promotion studies [32]. The model applies a combined schema, including the; ‘Type of Evidence Schema’, [33] ‘The Health Gains Notation framework’, [30, 31] and the ‘Cochrane Health Promotion and Public Health Field (CHPPHF) quality assessment screening questions for quantitative and qualitative studies [30, 32]. The CHPPHF tool assessed the quantitative studies for internal and external validity and rated the following criteria; allocation bias, selection bias, blinding, data collection methods, consent rate, statistical analysis and intervention integrity [34].

To be specific for the research area, the quality appraisal tools were modified to assess bias by reviewing the additional study characteristics; study type, multi-school sites, volunteer program, control/comparison groups, intervention parameters, survey methods and potential for confounders. In addition, the authors (GJ, FCW & KF) considered the outcomes, study length and follow-up period (longitudinal or short-term study) and how this might impact on the significance and generalisability of any findings.

The quality screening process led to 40 studies being classified as ‘moderate’ and therefore were well reported but the study designs could have been better by applying; larger sample sizes, achieving higher consent/participant rates, reducing risk of selection and recall bias, multi-site programs, applying control groups, or being multi-strategy comparison programs [12, 35–73].

However, due to the nature of the studies, with the majority being descriptive or observational studies and none being randomised control studies (RCT); ‘moderate’ was considered in this review as an acceptable standard. Almost all the studies suffered from selection bias as most rural programs were voluntary and many of the retrospective surveys would have encountered recall bias. No studies were classified as ‘very strong’, due to a lack of randomisation; however, six were assigned into the ‘strong category’ [74–79] due to their large sample sizes, or multi-site population, high consent or participation rates, clear reporting, control of potential rural confounders through statistical analysis, longitudinal reporting and use of control or applicable comparison groups.

Sixteen studies were deemed “weak”, for reasons such as; relatively poor response rates, small sample size, high risk of bias, lack of statistical analysis to control for potential confounders and not using control or comparison groups [80–95].

Additional file 2: Appendix S2 provides a detailed analysis summary of the quality review process for the 62 studies. This was a working table to summarise and evaluate each manuscript through a systematic process.

## Results

This section presents the trends in the methodological characteristics of the studies, and the outcome themes identified from the data extraction process. Additional file 1: Appendix S1 provides the reader with more specific summary information of the individual studies characteristics and outcomes.

### Methodological characteristics

Table 2 provides a quantitative summary of the selected studies methodological characteristics and key aspects of the table are discussed below.

**Table 2:** The methodological characteristics of the medical education articles

Characteristic	Ref Number	Frequency of Papers (Total papers = 62)
Location		
United States	[50–55, 60–62, 64, 65, 72, 75–77, 93–95]	18
Australia	[12, 35–49, 63, 67–71, 73, 78–81, 84–90, 92]	35
New Zealand	[82, 91]	2
Thailand	[74]	1
Canada	[56, 58, 59, 66, 83]	5
Africa	[57]	1
Program Classification Type (note programs can be in multi-classifications if they are comparison programs		
Rural Clinical Placement Programs (RCP)	[35–38, 44, 46, 56, 58, 59, 64–66, 68, 71, 73–75, 81–84, 86, 89–91, 93, 95]	27
Rural Clinical Placement Programs with specific rural, academic teaching emphasis (RCP + Edu)	[12, 50–55, 61, 62, 76, 80, 94]	12
Rural Clinical Schools Program (RCSP)	[39–45, 47–49, 57, 58, 60, 63, 64, 67, 69, 70, 72, 77–79, 85, 87, 88, 92]	26
Single/multi-institution		
Single medical school	[12, 36, 39–47, 49–56, 60–63, 68, 69, 72, 73, 76, 78, 80–89, 93–95]	42
More than one medical School	[35, 37, 38, 48, 57–59, 64–67, 70, 71, 74, 75, 77, 79, 90–92]	20
Length of Rural Clinical Placement (RCP)(note a program may have multiple durations)		
Less than 4 weeks	[35, 38, 46, 76, 80, 95]	6
4 weeks and up to 12 weeks	[12, 38, 44, 46, 50–52, 54, 58, 66, 91]	11
More than 12 weeks but less than 24	[38, 46, 52, 53, 58, 62, 94]	7
6 to 12 months	[36–38, 46, 55, 58, 61, 68, 73, 75, 82, 84, 86, 89, 93, 94]	16
2 years plus	[46, 56, 74, 75, 81, 83]	6
Unclassifiable/no set Placement length	[38, 64, 65, 90, 71]	5
Length of time in an RCSP evaluated program (note a program may be looking at multiple durations)		
Less than 4 weeks	[45, 92]	2
4 weeks and up to one 12 weeks	[70, 88, 92]	3
More than 1 term (12 weeks) and up to 1 year	[40–42, 44, 48, 49, 58, 63, 67, 69, 78, 79, 88, 92]	14
More than a year in a RCS	[40, 42, 43, 49, 57, 60, 69, 72, 77, 79, 85, 87]	12
Specific placement length not reported	[47, 64]	2
Objective of Intervention (simplified to rural intention or long-term follow-up for each study)		
Increase intention to practice rurally	[12, 35, 37, 38, 42, 45, 70, 80, 81, 85, 88–92]	15
Follow up to identify if a rural clinical intervention impacted on rural workforce participation	[36, 39, 41, 43, 44, 46, 49, 51, 53–67, 69, 71–75, 77–79, 83, 84, 86, 87, 94, 95]	38
Intention and follow up for actual rural location	[40, 47, 48, 50, 52, 68, 76, 82, 93]	9
Timing of the Evaluation		
Pre-and post-intervention Survey	[12, 37, 38, 42, 45, 73, 80, 81, 85, 90]	10
Post intervention survey Only (no follow up tracking)	[70, 88, 89, 91, 92]	5
Pre, post and follow-up post-graduation tracking	[40, 47, 50, 52, 68, 71, 74, 76, 93]	9
Post survey and post follow up tracking	[35, 36, 39, 44, 48, 56, 73, 82, 86]	9
Graduate Follow up Tracking only	[41, 43, 46, 49, 51, 53–55, 57–67, 69, 72, 75, 77–79, 83, 84, 87, 94, 95]	30
Comparison/Control Groups		
Studies using a control	[36, 50, 52–55, 57–60, 62, 63, 65, 66, 69, 72, 74, 77–79, 81, 83, 93, 95]	24
No control/comparison group	[12, 35, 37, 40, 42, 45, 48, 67, 70, 80, 84–86, 88, 89, 94]	16
Comparison groups but no non-intervention control group	[38, 39, 43, 44, 46, 56, 64, 68, 71, 73, 75, 82, 87, 90–92]	16
Comparison groups which include a control group	[41, 47, 49, 51, 61, 76]	6
Random allocation of students to intervention	–	0
Volunteer Status for participation in the Rural Program/School/Immersion		
Volunteer Program	[35–37, 39–43, 46–52, 54–56, 58–64, 66–73, 76–79, 81–95]	52
Non-Volunteer	[12, 45, 53, 57, 74, 80]	6
N/A/multiple programs/unknown	[38, 44, 65, 75]	4
Data Collection Method (studies may use more than one method)		
Questionnaire/Survey	[12, 35, 38–40, 42–45, 47–50, 52, 56, 58, 59, 61, 64, 67–71, 73, 76, 79–82, 84–92, 95]	40
Interviews	[37, 40, 61, 62, 82, 84, 92, 95]	8
Medical Schools Outcomes Database (MSOD) (National)	[38, 43, 48, 68, 70, 71, 90]	7
MUSOM Alumni Association database, Residency, Match Program, The American Board of Medical Specialties	[51, 52, 55]	3
Alumni Records (Local)	[35, 40, 44, 46, 49, 50, 52, 53, 55, 57, 60, 62, 63, 66, 69, 72, 74, 76, 78, 79, 83, 86, 93–95]	25
Other databases (Local)	[40, 46, 51, 53, 55, 59, 60, 62, 65, 74, 76, 82–84, 93, 94]	16
AHPRA (National)	[41, 43, 63, 69, 78, 79]	6
Internet/email/Phone	[35, 39, 40, 43, 51, 52, 64, 69, 74, 76, 82, 84, 86, 88, 94, 95]	16
American association Masterfile (AMA) (National)	[50, 54, 55, 60, 61, 65, 72, 75–77]	10
Focus Groups	[37, 80, 89]	3
Methods of Quantitative Analysis		
Accounted for rural confounders/impacting factors through statistical analysis	[12, 36, 38–42, 44, 45, 47–49, 51–53, 55, 56, 58–66, 68–79, 81–85, 87–91, 93, 95]	50
Did not include rural confounders/impacting factors in statistical calculations (i.e. did not adjust) Inferential statistics, unadjusted)	[35, 37, 43, 46, 50, 54, 57, 67, 80, 86, 92, 94]	12
Sample Size (Includes both intervention/control/comparison group if applicable)		
Less than 100	[37, 50, 73, 80–82, 84–89, 92, 93]	14
100–500	[12, 35, 39, 40, 42, 44, 45, 47–49, 54, 56–59, 62, 67, 68, 70, 83, 90, 91, 94, 95]	24
500+	[36, 38, 41, 43, 46, 51–53, 55, 60, 61, 63–66, 69, 71, 72, 74–79]	24
Consent or Participant Rate (Depending on the study/type of study and reporting)		
Below 50%	[35, 47, 58, 59, 69, 71, 73, 79, 81, 88, 91]	11
50–80%	[39, 40, 44, 45, 48, 49, 51, 56, 64, 67, 68, 70, 80, 82, 84–86]	17
81–100%	[12, 36, 38, 41–43, 46, 50, 52–55, 57, 60–63, 65, 66, 72, 74–78, 83, 87, 89, 92–95]	32
Not reported	[37, 90]	2

#### Classification type

The studies were separated into three classifications. The first was a Rural Clinical Placement (RCP) program (twenty seven studies) [35–38, 44, 46, 56, 58, 59, 64–66, 68, 71, 73–75, 81–84, 86, 89–91, 93, 95]. These included any rural clinical placement programs where participants were from a metropolitan based school (the school or study is not based in a rural location and is not classed as part of a rural clinical school).

The second classification were Rural Clinical Placement Programs (same criteria as a RCP) with an additional rural education component (RCP + Education) (12 studies) [12, 50–55, 61, 62, 76, 80, 94]. The additional rural educational component was often rural cultural training, Aboriginal culture and/or extended general rural employment and community information.

Rural Clinical School Programs (RCSP) were the final classification (twenty-six studies) and this includes involving schools located within a geographic rural location, and/or studies/schools self-identified as part of a rural clinical school (irrespective of their location.) (For example, a RCS may be located in a metropolitan location but may be termed an RCSP, as they have rural campuses and rural initiative programs) [39–45, 47–49, 57, 58, 60, 63, 64, 67, 69, 70, 72, 77–79, 85, 87, 88, 92]. One study directly compared a RCSP to a RCP [44] and two studies looked at large populations that related to both RCP and RCSP programs, hence these programs have been placed in both classifications [58, 64].

#### Study location

Thirty five articles were from Australia, [12, 49, 63, 67–71, 73, 78–81, 84–90, 92] eighteen from the United States [50–55, 60–62, 64, 65, 72, 75–77, 93–95] five from Canada [56, 58, 59, 66, 83] two from New Zealand [82, 91], and one each from Thailand [74], and Africa [57].

Forty-two of the studies were designed and implemented within a single medical school (rather than a partnership with multiple schools) [12, 36, 39–47, 49–56, 60–63, 68, 69, 72, 73, 76, 78, 80–89, 93–95], while twenty programs involved multiple medical schools [35, 37, 38, 48, 57–60, 64–67, 70, 71, 75, 77, 79, 90–92].

#### Participants, consent rate and volunteer status

The most commonly reported sample size was split between 500plus [36, 38, 41, 43, 46, 51–53, 55, 60, 61, 63–66, 69, 71, 72, 74–79], (24 studies) and between 100 and 500 participant sample size (24 studies) [12, 35, 39, 40, 42, 44, 45, 47–49, 54, 56–59, 62, 67, 68, 70, 83, 90, 91, 94, 95]. There was a wide spread in sample size from small to large population size programs across the literature. More than 50% of studies reported high participant or consent rates of 81–100% [12, 36, 38, 41–43, 46, 50, 52–55, 57, 60–63, 65, 66, 72, 74–78, 83, 87, 89, 92–95].

More than three quarters of the programs were volunteer programs, which involved participants either applying for a program or agreeing to participate upon invitation [35–37, 39–43, 46–52, 54–56, 58–64, 66–73, 76–79, 81–95].

#### Control and comparison groups

Less than half [24] of the studies were identified as having a control group [36, 50, 52–55, 57–60, 62, 63, 65, 66, 69, 72, 74, 77–79, 81, 83, 93, 95], however, 46 studies reported using a control group, and or a comparison group, or both [36, 38, 39, 41, 43, 44, 46, 47, 49–66, 68, 69, 71–79, 81–83, 87, 90–95]. Therefore, 16 studies did not report using a control group or comparison group [12, 35, 37, 40, 42, 45, 48, 67, 70, 80, 84–86, 88, 89, 94]. A control group was classified by being a control against the intervention, thereby receiving no intervention and used as a baseline to assess the effect of the intervention. A comparison group was identified as a separate comparison intervention rather than a control cohort. An example of a comparison group, would be a study comparing international medical participants with the intervention of a rural placement program.

#### Research methods

Questionnaire survey format (40 studies) [12, 35, 38–40, 42–45, 47–50, 52, 56, 58, 59, 61, 64, 67–71, 73, 76, 79–82, 84–92, 95] and localised alumni school records (25 studies) [35, 40, 44, 46, 49, 50, 52, 53, 55, 57, 60, 62, 63, 66, 69, 72, 74, 76, 78, 79, 83, 86, 93–95] were the most commonly used methods of evaluation. Twenty-three studies were identified as using national registration databases including the; Australian Health Practitioner Regulation Agency (AHPRA) [41, 43, 63, 69, 78, 79] the American Medical Association (AMA) Physician Masterfile [50, 54, 55, 60, 61, 65, 72, 75–77], and the Medical Schools Outcomes Database (MSOD) [38, 43, 48, 68, 70, 71, 90].

Post graduate follow up/survey tracking, was the most commonly reported evaluation timing (30 studies) [41, 43, 46, 49, 51, 53–55, 57–67, 69, 72, 75, 77–79, 83, 84, 87, 94, 95], and ten studies used pre-and post-surveys with no graduate tracking [12, 37, 38, 42, 45, 73, 80, 81, 85, 90]. Only nine studies reported completing pre, post intervention surveys and follow up tracking of the graduates [35, 36, 39, 44, 48, 56, 73, 82, 86].

#### Data analysis

The quantitative analysis methods were classified; either by adjusting for rural confounders/predictors within the statistical analysis model, or not adjusting for them. Over three quarters of the studies were noted as adjusting for one or more potential rural confounders within the statistical analysis methods [12, 36, 38–42, 44, 45, 47–49, 51–53, 55, 56, 58–66, 68–79, 81–85, 87–91, 93, 95].

#### Placement/program duration

The duration period of the rural interventions (placements and programs) were divided by RCP and RCP + Edu programs classifications combined, and RCSP.

For the RCPs combined, the most commonly reported period (16 studies) were six to 12 months [36–38, 46, 55, 58, 61, 68, 73, 75, 82, 84, 86, 89, 93, 94], and 11 studies were between four weeks and up to 12 weeks [12, 38, 44, 46, 50–52, 54, 58, 66, 91].

For RCSP the most common program length was 12 weeks and up to a year (14 studies) [40–42, 44, 48, 49, 58, 63, 67, 69, 78, 79, 88, 92], and more than a year (12 studies). [40, 42, 43, 49, 57, 60, 69, 72, 77, 79, 85, 87] It should be noted that multiple programs ran multiple program length interventions in all classifications.

### Key outcome themes identified

Table 3 presents the key outcome themes identified within the literature, and the outcomes are also separated by the study strength and rural clinical program classification.

**Table 3:** The Key outcome themes by classification type and study quality

OUTCOME THEMES	STUDY CLASSIFICATION												
	RCP (CLASS 1 Total)	References (CLASS 1)			RCP + EDU (CLASS 2 Total)	References (CLASS 2)			RCSP (CLASS 3 Total)	References (CLASS 3)			TOTAL ALL CLASSIFICATIONS (Removed duplicates)
		Study Quality				Study Quality				Study Quality			
		Weak	Mod	Strg		Weak	Mod	Strg		Weak	Mod	Strg	
Workforce Intentions/Outcomes													
Increased association with graduates working in a rural location (reported numbers of graduates going onto work in a rural location)	**21**	[82]	[36]	[74]	**9**	[94]	[50]	[76]	**15**		[39]	[77]	**43**
		[83]	[46]	[75]			[51]				[40]	[78]	
		[84]	[54]				[52]				[41]	[79]	
		[86]	[56]				[53]				[43]		
		[91]	[58]				[61]				[57]		
		[93]	[59]				[62]				[58]		
		[95]	[64]				[65]				[60]		
			[65]								[63]		
			[66]								[67]		
			[68]								[69]		
			[71, 73]								[70]		
											[72]		
Increased Rural Intentions/likelihood to work rurally	**13**	[81, 82]	[35]	[75]	**3**		[12, 50]		**13**	[85]	[39]		**28**
		[86, 89]	[36]				[61]			[87]	[40]		
		[90, 91]	[37]							[88]	[42]		
			[38]							[92]	[44]		
			[44]							[93]	[47]		
			[71]								[48]		
											[49]		
											[70]		
Increased student interest in rural health medicine	**10**	[81]	[35]	[75]	**4**	[80]	[12]	[76]	**5**	[85]	[42]		**20**
		[82]	[37]				[57]			[92]	[45]		
		[84]	[70]								[49]		
		[86]											
		[87]											
		[88]											
		[89]											
Long term retention of graduates reported	**3**			[56]	[74]	**3**		[50]		**1**		[72]	**7**
				[57]				[54]					
								[62]					
Reported low retention rates of rural employed grads and a highly mobile workforce	**0**				**0**				**1**		[67]		**1**
Intervention did not show a positive association with graduates choosing rural employment	**2**	[81]	[64]		**0**	–			**4**	[87, 88]	[47]		**5**
											[64]		
The Impact of Rural Background													
Rural Background is an important predictor toward rural intentions or rural employment	**10**	[82]	[38, 58]	[74]	**6**		[51]	[76]	**11**		[42]	[77]	**25**
		[84]	[64, 66]				[52]				[43]	[79]	
		[91]	[68, 71]				[54]				[49]		
							[55]				[58]		
							[61]				[60]		
											[64]		
											[69]		
											[70]		
											[72]		
Rural background of a participant is a more predictive factor of rural intentions than an educational clinical placement intervention	**3**		[37, 38]		**0**				**5**		[43]	[77]	**7**
			[64]								[49]		
											[64]		
											[70]		
Intervention outcomes independent of the rural background and therefore clinical rural experience more significant	**4**	[91]	[36, 58]		**3**		[12, 52]	[76]	**6**	[85, 87]	[39]	[78]	**13**
			[73]							[88]	[41]		
Other Identified Factors/Predictors of Rural Employment													
Intentions at the start of medical training is a greater predictor of rural intentions than a rural intervention	**4**	[81]	[38, 71]		**2**		[53]	[76]	**3**		[40]		**9**
		[86]									[42]		
											[43]		
Increased period of exposure (placement or RCS) led to an increased association with rural intentions/employment	**7**	[81]	[35, 36]		**0**				**9**	[92]	[39]	[79]	**15**
			[38, 46]								[40]		
			[58, 66]								[42]		
											[49]		
											[58]		
											[60]		
											[69]		
Generalist intentions were a key predictor of rural intentions/rural work	**3**	[81]	[35, 37]		**0**	–			**5**		[48]	[79]	**8**
											[49]		
											[67]		
											[70]		
Family medicine associated with rural clinical interest/employment	**2**		[59]	[75]	**8**		[50, 51]		**2**		[58]		**12**
							[52, 53]				[60]		
							[54, 55]						
							[61, 62]						
Primary care associated with rural clinical interest/employment	**0**				**5**		[50, 51]		**5**		[67]	[75]	**10**
							[52, 53]				[72]	[76]	
							[62]					[77]	
Scholarship (Bonded)	**1**		[71]		**0**				**2**		[69]	[79]	**3**
Being in graduate entry program negatively associated with working rurally	**1**		[71]										**1**
Having dependent children negatively associated with working rurally	**1**		[71]										**1**
Further Research													
Long term follow-up of the study required	**9**	[82]	[36, 37]		**3**	[94]	[55, 62]		**11**	[85, 87]	[40]		**22**
		[86]	[44, 68]							[88]	[42]		
		[90]	[71]								[43]		
		[91]									[44]		
											[45]		
											[49]		
											[57]		
											[70]		
Exploration is needed of the specific characteristics of RPs that are associated with students’ intended location of future medical practice.	**3**	[90]	[38]		**2**		[54]		**4**		[39]		**9**
		[91]					[55]				[41]		
											[42]		
											[43]		

Note: The summary totals for Table 3 removed duplicates (i.e. where a paper comes under multiple classifications, which occurred in several comparison studies) we counted the identified outcome once in the total column

The bolded numbers represent the total ref for each classification

#### Workforce outcomes

Across all the studies, the increased numbers of graduates (or increased association) working in a rural location (43 papers) [36, 39–41, 43, 46, 50–54, 56–79, 82–84, 86, 91, 93–95] was the most reported outcome, followed by increased rural intentions/or an increased likelihood to work rurally (28 papers) [12, 35–40, 42, 44, 47–50, 61, 70, 71, 75, 81, 82, 85–93]. Twenty papers reported increased student interest in rural health medicine [12, 35, 37, 42, 45, 49, 57, 70, 75, 76, 80–82, 84–89, 92]. Seven studies reported long term retention of graduates employed in a rural location [50, 54, 56, 57, 62, 72, 74]. Five studies did not report a positive association with graduates choosing rural employment [47, 64, 81, 87, 88].

Looking specifically at the six strongest quality studies, as deemed through the quality review, all reported increased association with rural employment [74–79]. and only one of these studies reported positive rural intentions [75] as the studies were focused on workforce outcomes. Two of the strong studies reported long term retention of the graduates [72, 74] while none of the weaker studies reported positive rural workforce retention rates.

#### The rural background factor

Forty percent of the studies (25 studies) acknowledged rural background as an important predictor of rural workforce intentions or rural employment [38, 42, 43, 49, 51, 52, 54, 55, 58, 60, 61, 64, 66, 68–72, 74, 76, 77, 79, 82, 84, 91]. Thirteen studies [12, 36, 39, 41, 52, 58, 73, 76, 78, 85, 87, 88, 91], reported that the positive rural intentions/workforce outcomes reported were independent of the students’ rural background and that a rural clinical experience itself is a more significant predictor of rural intentions. However, seven studies contradicted this relationship and reported that rural background is a greater predictive factor of rural intentions [37, 38, 43, 49, 64, 70, 77].

Four of the strongest studies identified rural background as an important predictor of rural employment [74, 76, 77, 79] and one of the group reported that rural background is a more predictive factor of rural intentions than an educational placement experience [77].

#### Additional potential predictors of rural employment

An identified theme reported in nine studies, was that students’ rural intentions prior to the start of medical training is a better predictor of rural intentions than a rural intervention [38, 40, 42, 43, 53, 71, 76, 81, 86]. Eight studies reported that generalist clinical intentions by students were a key predictor of rural intentions [35, 37, 48, 49, 67, 70, 79, 81]. Fifteen studies reported that increasing the length of rural exposure (when they had comparison placement program lengths) led to an increased association with either rural intentions and/or rural employment [35, 36, 38–40, 42, 46, 49, 58, 60, 66, 69, 79, 81, 92].

Other potential predictors of rural employment or associated factors were identified as; family medicine [50–55, 58–62, 75] and primary care [50–53, 62, 67, 72, 75–77], and a recent 2016 paper reported to be the first study to demonstrate that being in a graduate entry program or having dependent children, is negatively associated with working rurally [71].

#### Further research required

Twenty two papers concluded that long term program follow up is required to draw stronger evidence of the impact of rural placements on workforce outcomes [36, 37, 40, 42–45, 49, 55, 57, 62, 68, 70, 71, 82, 85–91, 94]. Nine studies reported of the need to explore the specific characteristics of rural placement programs that are associated with students’ intention to work rurally [38, 39, 41–43, 54, 55, 90, 91].

### Outcome themes by study classification

Table 3 also shows that the trends in the outcomes were similar across the study classifications, with increased association with rural employment and positive rural intentions trending similarly. The RCP classification had the highest number of studies reporting a positive rural association (21studies) [36, 46, 54, 56, 58, 59, 64–66, 68, 71, 73–75, 82–84, 86, 91, 93, 95] compared to 15 studies for the RCSP. [39–41, 43, 57, 58, 60, 63, 67, 69, 70, 72, 77–79] RCP + Edu had the lowest reported, but there were fewer studies identified in this classification (9 studies) [50–53, 61, 62, 65, 76, 94].

Other outcomes also trended similarly between the classifications, with increased intervention exposure leading to increased association with rural intentions/rural employment in the RCP [35, 36, 38, 46, 58, 66, 81] (seven studies) and RCSP [39, 40, 42, 49, 58, 60, 69, 79, 92] (nine studies), however none were reported for the RCP + Edu.

Of the six strongest quality studies, three were RCSP’s [77–79], two RCPs [74, 75] and one RCP + Edu [76].

## Discussion

This review has provided a timely update on the literature related to rural outreach schemes in medical education and workforce outcomes. The review has presented the methodological characteristics of the identified evidence base, identified workforce outcome themes and reviewed the quality of the literature. By drawing out the methods the studies applied, it has allowed program implementation and evaluation recommendations to be made, and the extracted workforce outcome themes provide a detailed presentation of the key workforce factors related to the identified rural initiatives.

This discussion will present the methodological characteristics of the literature, the workforce outcome themes and identified workforce factors; and then compares the findings with other current review evidence, in order to consider implications and make recommendations.

### Methodological characteristics

The high number of Australia and US studies supports Dolea, Stormont and Braichet [17] who reported that workforce studies come from high income countries. An explanation for the prominence of studies in Australia may be due to the government focus on addressing rural workforce shortages through the RUSC program and significant government investment in undergraduate clinical training in 2008 [25, 26].

Much of the evidence consists of descriptive cross-sectional studies, longitudinal tracking projects and a number of large cohort studies. No RCTs are identified, and while this methodological standard would reduce selection bias, it would be difficult logistically; and ethically challenging to make educational interventions mandatory and assign students to specific groups for sampling robustness [96]. Victora, Habicht and Bryce [97] reported that RCTs are often inappropriate for the assessment of the impact of large scale complex public health interventions and evidence-based public health must go beyond the RCT model.

Most studies involved single University/site programs and were often small to moderate sample sizes of the intervention group, limiting the power and generalisability of the findings. There was a lack of pre-questionnaires to measure important attributes such as rural background, rural intentions/interest and career intent, prior to intervention. However, a number of studies had pre-admission criteria to the programs and collected important participant information from alumni records. Pre-and post-survey data and post-graduate workforce follow-up is, in general, a more robust method of determining longitudinal change in student attitudes and provides important participant information to assist in controlling for potential rural confounders. It is noted however in the larger retrospective programs looking at workforce trends across large populations and multiple programs, that pre and post in-depth survey evaluation is not always applicable or possible.

A considerable number of studies did not use control groups, which are considered essential to Cochrane-type evidence-based research analysis to effectively measure change and the magnitude of effect, and to minimise the impact of potential confounding variables except for the independent variable [17]. In addition, the majority of the programs involved voluntary participation and this can lead to self-selection bias, with volunteer respondents more likely to favour rural employment and respond positively to the anticipated rural workforce outcomes. In addition, it was noted that the majority of studies did not provide information on the specific evaluation tools applied, such as questionnaire format/style and this limited this reviews ability to report on evaluation methods applied, such as the application of Likert Scales, or questionnaire structure. Furthermore, a number of studies did not provide a detailed explanation of the intervention and specific program aspects, which is important to assess, or for successful programs to consider adopting. This finding supports prior evidence which has reported that rural immersion research has been mainly focussed on outcomes, with limited description of aspects such as program design and student selection [27, 98, 99].

Longitudinal tracking programs were commonly reported in the review, however the length of the follow up varies considerably, with some looking at intern choices, directly after graduation and others tracking longer term workforce outcomes. This finding supports a 2018 scoping review of Australian immersion programs, which stated that the time point at which the follow up of graduates working location occurs is important. The study provided an example, as it stated internships do not reflect ‘graduate choice’ of employment location, but rather it is a matching allocation process based on student and hospital preference and it occurs within a competitive state-based system [100]. Furthermore this review identifies studies are often only measuring workforce outcomes at one point in time and therefore there is a lack of rural workforce retention data. The need for longitudinal programs is supported by Humphreys et al. [101] who comment that well conducted, purpose built, ongoing studies with longitudinal data are required to produce robust medical workforce planning evidence. Pharwaller et al. [31] stated there is a need in rural workforce programs for multifaceted strategies organised within coherent longitudinal programs.

The most reported program duration (placement or RCSP) was between six months and one year. The number of year-long programs in Australia can be attributed to Australia implementing a policy in 2000, to fund medical schools to select 25% of students from a rural background, and also for 25% of students to participate in at least one year of rural clinical training [102, 103]. A number of studies in this review report that increasing the length of the rural exposure increases the association of rural influence in terms of rural intentions/rural employment. Previous studies suggest that the ideal placement length and timing is still unknown and needs further exploration. The mixed lengths in this review support the need for further investigation, but the majority of evidence leans towards longer placements/programs (more than 6-12 months) being associated with positive rural workforce outcomes [27, 99, 104].

Ranmuthugala et al. [27] assert that studies fail to adjust for critical independent predictors of rural practice, and programs need to systematically investigate specific aspects of the rural experience to identify the factors that create a positive impact on trainee medical practitioners. An Australian review [103] states that it is challenging for research to isolate the independent causal effect of rural programs on rural employment. This review reports that improved data collection methods and statistical analysis in the control for potential predictors, is now occurring in a larger number of studies. However, within the literature there is little consistency in the application of these rural predictors and the statistical modelling applied, and it is proposed that identifying set specific predictors for future programs may produce more consistent and higher quality research.

### Key outcome themes and identification of potential rural workforce predictors

The positive rural workforce themes reported in this review supports a 2008 systematic review by Rabinowitz et al. [105] which identified a multi-fold increase in the rural physician supply and reported strong evidence that medical schools have significant potential to address the workforce mal-distribution. The study also stated the importance of identifying any positive workforce outcomes from effective programs, as even a small number of rural clinicians can have a critical impact on a rural location (due to ratio of clinician to rural population numbers), as one rural clinician can have a major impact on access to care for rural communities. They provide an example of the PSAP program which has on average 14 students participate per year but leads to 12% of rural family physicians in the state of Pennsylvania [105, 106]. The positive associations between rural programs and rural employment identified in this review, supports a 2018 review which reported that Australia’s immersion programs are moderately associated with increased supply of junior doctors into rural locations [100].

Rural background is deemed an important predictor of rural intentions and/or rural employment by more than a third of the studies. Furthermore, the majority of the highest quality studies deemed it a key predictor of rural intentions and or rural employment, and that it should be factored and considered within rural program planning. Two reviews state that being from a rural background is the strongest predictor of choosing a rural practice location [28, 107]. In addition, an independent Australian Government review recommended that students with a rural background should be preferentially offered places to improve health workforce outcomes [108]. However, a recent review comments that due to the lack of students from rural backgrounds this means that rural background recruitment should not be the primary policy for addressing rural workforce shortages, and interventions need to target all students [28]. Playford et al. [109] reported that more is needed than selecting students with a rural background and while a rural intent is important, combining with rural exposure during training increases the likelihood of rural employment [109].

It is clear that rural background is an important aspect for program planners to consider, however our review identifies there are other key potential rural predictors including; rural interest/intentions prior to the program, generalist practice intentions, an interest in primary care and family medicine, financial and rural bonded scholarships and importantly the type and quality of a rural immersion experience and its duration. The control for rural predictors is crucial and is supported by a recent (2017) evaluation of a rural clinical school program, which reported that rural background, rural intention and rural experience during medical school, all need to be incorporated within future workforce programs [109].

In addition, a 2017 systematic review identified an association between family medicine focus and primary care interest, with rural practice [28]. It is clear there are a wide range of program parameters to consider in the development of a rural program experience.

#### Study limitations and strengths

The initial searches in this study used key words, one of which led to 454,000 results. Multiple combinations of key words were therefore applied to increase specificity. It was expected that using educational search terms would lead to a high number of results as was encountered by Crampton, McLachlan and Llling [96], who reported that the balance between sensitivity and specificity is a complex challenge with modern systematic reviews. Also, a large search study base may lead to errors and the chance of missing relevant studies. However, the ease of use of modern databases and their comprehensive search engines means that relatively accurate systematic searches are possible. Multiple databases were searched, and findings were limited to published papers considering education evaluation programs related to rural intentions and workforce outcomes. The reference lists of all manuscripts analysed in the review were studied and applicable papers included to attempt to reduce the likelihood of missing relevant studies.

A strength of this review is that despite a large range of interventions, methods and quality of studies, the findings were generally similar and reported positive rural clinical experiences, increased rural intentions, and positive associations with increased rural employment. In addition, the quality of the interventions were assessed; and a general score was applied over a more specific system [32–34]. A further methodological strength of this review is that we used the PRISMA protocol instructions as a guide in the systematic review structure [29, 30]. In regards to the quality review, the authors would like to comment on the challenges of categorising such wide ranging studies within three broad categories and ascertaining the weight of certain study characteristics on the quality of a manuscript. The authors mitigated this challenge through a systematic transparent approach (Additional file 2: Appendix S2), discussions of contestable papers and attributed caution and lower categorisation when a manuscript appeared to cross multiple categories.

#### Implications

The breadth of different programs this review identifies, demonstrates uncertainty about the types of rural educational programs that are most effective in terms of placement length, timing and frequency, and that program planners are continuing to refine and build larger and more ambitious longitudinal research programs [27]. The review highlights some study design limitations being applied in these medical education programs, with a large number of studies not using control groups or pre-questionnaires to identify important participant characteristics and views on rural interest/employment, that could provide important data for statistical modelling. Furthermore, the review notes the lack of clarity on the potential predictors of rural employment that need to be effectively controlled for and managed, and the lack of consistency in the application of these potential predictors across the literature. The majority of the evidence focusses on descriptive, cross sectional and cohort research findings of any significant positive associations, rather than conclusive causal factors in health workforce predictions. Nevertheless, it can be concluded that well designed and financially supported rural clinical placement programs and rural clinical schools do have a positive association with rural practice location, and that these programs warrant continued investment and further longitudinal review, with longer term retention information of graduates’ employment history a key area that requires further investigation.

It is only with the availability of more rigorous evidence and sufficient political commitment that we will be able to address the pressing issue of equitable healthcare delivery in rural locations and identify winning strategies to guide future practice and policy. Based on the reflection of the literature a number of key recommendations have been made below.

#### Key recommendations

- Future studies should focus on more methodologically rigorous longitudinal studies with measurements at multiple time points and report on graduate rural employment retention, with an aim to, isolate and measure the impact of the individual predictors that influence the career choice of medical students/graduates.
- In-depth qualitative research may further assist in exploring the intrinsic and extrinsic predictors driving rural intentions and actual rural workforce outcomes. This additional information would help to contextualise the larger scale quantitative data being reported.
- Programs should incorporate pre and post surveys to assess the change in participants after the program intervention, and to collect important participant characteristic information (e.g. pre-program rural interest and rural intentions).
- Future programs should analyse associations and control for potential rural workforce predictors through statistical analysis (e.g. multivariate statistical modelling). Programs need to be more consistent in the rural predictors (confounders) they aim to control and should include; rural background, rural experiences prior to the program and pre-placement/program rural intentions. In addition, programs should consider controlling for and further investigating the impact of potential confounders including; student’s interest in primary care, family medicine and generalist practice, bonded scholarships, graduate schemes and graduates with child dependency.
- All future studies should apply control (preference on specific control groups) or comparison groups to effectively measure the change and impact of an intervention.
- Rural background is a significant, independent predictor of workforce choices and should be considered within all rural placement research. Considerable evidence supports selecting rural background students for medical schools and multiple studies have identified combining rural background students with a rural educational initiative as a more powerful means of encouraging rural employment. However, given the dearth of rural background students compared to metropolitan background students, rural educational initiatives need to also target both groups, especially as a number of studies have identified that an effective rural education intervention, is an independent predictor of rural employment outcomes.
- Further investigation is required to ascertain the optimum program period length; however, the strongest evidence in medicine trends toward periods of six months or longer to provide a significant rural experience.
- Due to the large number of single site developed and managed RCP and RCSP identified in this review, it is advised that medical institutions consider greater collaboration between schools and other partners; in order to develop multi-site programs to increase the impact, power and generalisability of the evidence base. Larger scale multi-site programs should provide more powerful data to help inform national policy.

## Conclusions

The review’s detailed presentation of the studies methodological characteristics, outcomes and recommendations, makes it useful to inform future research on rural placement programs. Investigators need to record the factors driving the success of programs through applying pre-and post-placement surveys, control groups, and large scale methodologically rigorous longitudinal cohort studies which look at workforce outcomes at multiple time-points. Programs should also consider partnering up to expand beyond single institution programs to enhance the power and generalisability of the evidence. In addition, explorative and in-depth qualitative research should be considered to explore the predictive/causal factors within successful programs.

The goal must be to establish a successful and repeatable design model for rural clinical programs that can be replicated and implemented on an international or national basis. In addition, policy advisors and future placement program developers should consider the evidence presented in this review, as a guide of what is working and where the evidence base is currently positioned.

## Additional files

Additional file 1: Appendix S1. A summary of the 62 selected studies. Provides the reader with summary information of the 62 selected studies specific characteristics and outcomes. (DOCX 77 kb)

Additional file 2: Appendix S2. A summary analysis of the quality review process for the 62 selected studies. Provides a detailed analysis summary of the quality review process for the 62 studies. This is a working table to summarise and evaluate each manuscript through a systematic process. (XLS 263 kb)

## Abbreviations

AHPRA: Australian Health Practitioner Regulation Agency
AMA: American Medical Association
CHPPHF: Cochrane Health Promotion and Public Health Field
MSOD: Medical Schools Outcomes Database
PRISMA: Preferred Reporting Items for Systematic reviews and Meta-Analysis
RCP + Edu: Rural Clinical Placement with an additional educational component
RCP: Rural Clinical Placement
RCS: Rural Clinical Schools
RCSP: Rural Clinical School Programs
RCTs: Randomised Control Trials
RUSC: Rural Undergraduate Support and Co-ordination Program
US: United States

## References

[1] Australian Government, The Department of Health. In: National strategic framework for rural and remote health. 2016. http://www.health.gov.au/internet/main/publishing.nsf/Content/national-strategic-framework-rural-remote-health. Accessed 1 July 2017.

[2] The Sentinel Watch American Sentinel University. In: Health disparities continue to plague rural areas. 2016. http://www.americansentinel.edu/blog/2016/07/26/health-disparities-continue-to-plague-rural-areas/. Accessed 1 July 2017.

[3] Laven GA, Laurence COM, Wilkinson D, Beilby JJ. National Rural Health Conference. In: Using the Australian rural background study to inform rural and remote multidisciplinary health workforce planning research. Central to health: sustaining well-being in remote and rural Australia. Alice Springs, NT: Proceedings of the 8th National Rural Health Conference. 2005. https://ruralhealth.org.au/8thNRHC/Papers/laven,%20gillian.pdf. Accessed 1 July 2017.

[4] Ricketts TC (2005). Workforce issues in rural areas: a focus on policy equity. Am J Public Health.

[5] Renner DM, Westfall JM, Wilroy LA, Ginde AA. The influence of loan repayment on rural healthcare provider recruitment and retention in Colorado. Rural Remote Health 2010;10:1605. http://www.rrh.org.au/publishedarticles/article_print_1605.pdf. Accessed 1 July 2017. 21070088

[6] World Health Report. In: The world health report 2006, working together for health. World Health Organization. 2006. http://www.who.int/whr/2006/en/. Accessed 1 July 2017.

[7] Rosenblatt R (2004). A view from the periphery, health care in rural America. N Engl J of Med.

[8] Australian Institute of Health and Welfare. Health and community services labour force. National health labour force. Australian Government. Canberra. 2009. https://www.aihw.gov.au/getmedia/cb651a4b-1c3b-4199-bdd9-c8a68d8b61ea/hwl-43-10677.pdf.aspx?inline=true. Accessed 1 July 2017.

[9] Serneels P, Lindelow M, Montalvo J, Barr A (2007). For public service or money: understanding geographical imbalances in the health workforce. Health Policy Plan.

[10] Lehmann U, Dieleman M, Martineau T (2008). Staffing remote rural areas in middle- and low-income countries: a literature review of attraction and retention. BMC Health Serv Res.

[11] Bazen JJ, Kruger E, Dyson K, Tennant M. An innovation in Australian dental education: rural, remote and indigenous pre-graduation placements. Rural Remote Health 2007;703. http://www.rrh.org.au/journal/article/703. 17696758

[12] Critchley J, DeWitt DE, Khan MA, Liaw S. A required rural health module increases students’ interest in rural health careers. Rural Remote Health 2007;7:688. http://www.rrh.org.au/articles/subviewnew.asp?ArticleID=688. Accessed 1 July 2017. 17547493

[13] Laven G, Wilkinson D (2003). Rural doctors and rural backgrounds: how strong is the evidence? A systematic review. Aust J Rural Health.

[14] Woloschuk W, Tarrant M (2002). Does a rural educational experience influence students' likelihood of rural practice? Impact of student background and gender. Med Educ.

[15] Capstick S, Beresford R, Gray A (2008). Rural pharmacy in New Zealand: effects of a compulsory externship on student perspectives and implications for workforce shortage. Aust J Rural Health.

[16] Mathews M, Rourke JTB, Park A (2008). The contribution of Memorial University’s medical school to rural physician supply. Can Journal Rural Med.

[17] Dolea C, Stormont L, Braichet J. Evaluated strategies to increase attraction and retention of health workers in remote and rural areas. Bull World Health Organ 2010; 88:379–385. http://www.who.int/bulletin/volumes/88/5/09-070607.pdf. Accessed 1 July 2017. 10.2471/BLT.09.070607 PMC2865654 20461133

[18] Health Workforce Australia. In: Health Workforce 2025 – Doctors, nurses and midwives – volume 1. 2012. https://submissions.education.gov.au/forms/archive/2015_16_sol/documents/Attachments/Australian%20Nursing%20and%20Midwifery%20Accreditation%20Council%20(ANMAC).pdf. Accessed 1 July 2017.

[19] Hawthorne L (2012). International medical migration: what is the future for Australia?. Med J Aust Open.

[20] Daniels Z, VanLeit B, Skipper B, Sanders M, Rhyne R (2007). Factors in recruiting and retaining health professionals for rural practice. National Rural Health Association. J Rural Health.

[21] Russel JD, McGrail RM, Humphreys JS (2016). Determinants of rural Australian primary health care worker retention: a synthesis of key evidence and implications for policymaking. Aust J Rural Health.

[22] Sen Gupta TK, Murray RB, McDonell A, Murphy B, Underhill AD. Rural internships for final year students: clinical experience, education and workforce. Rural Remote Health 2008;8:827. http://www.rrh.org.au/publishedarticles/article_print_827.pdf. Accessed 1 July 2017. 18271675

[23] Playford D, Larson A, Wheatland B (2006). Going country: rural student placement factors associated with future rural employment in nursing and allied health. Aust J Rural Health.

[24] Grobler L, Marais BJ, Mabunda SA, Marindi PN, Reuter H, Volmink J. Interventions for increasing the proportion of health professionals practicing in rural and other underserved areas. Cochrane Database Syst Rev. 2009. Issue 1. Art. No.;CD005314. 10.1002/14651858.CD005314.pub2. 10.1002/14651858.CD005314.pub2 19160251

[25] Eley DS, Young L, Wilkinson D, Chater AB, Baker PG (2008). Coping with increasing numbers of medical students in rural clinical schools: options and opportunities. Med J Aust.

[26] The Department of Health. In: Appendix iii: History of commonwealth investment in the medical workforce. Review of Australian government health workforce programs. 2013. http://www.health.gov.au/internet/publications/publishing.nsf/Content/work-review-australian-government-health-workforce-programs-toc~appendices~appendix-iii-history-commonwealth-investment-medical-workforce.

[27] Ranmuthugala G, Humphreys J, Solarsh B, Walters L, Worley P, Wakerman J (2007). Where is the evidence that rural exposure increases uptake of rural medical practice?. Aust J Rural Health.

[28] MacQueen I, Maggard-Gibbons M, Capra G, Raaen L, Ulloa J, Shekelle P (2017). Recruiting rural healthcare providers today: a systematic review of training program success and determinants of geographic choices. JGIM.

[29] PRISMA 2009 Checklist. In: PRISMA-Statement. 2009. http://prisma-statement.org/documents/PRISMA%202009%20checklist.pdf. Accessed 1 July 2017.

[30] Moher D, Shamseer L, Clarke M, Ghersi D, Liberati A, Petticrew M et al. Preferred reporting items for systematic review and meta-analysis protocols (PRISMA-P) 2015: elaboration and explanation. Res Methods Reporting Br Med J 2015;349: 7647. https://systematicreviewsjournal.biomedcentral.com/articles/10.1186/2046-4053-4-1. 10.1136/bmj.g7647 25555855

[31] Pfarrwaller E, Sommer J, Chung C, Maisonneuve H, Nendaz M, Perron NJ et al. Impact of interventions to increase the proportion of medical students choosing a primary care career: a systematic review. J Gen Intern Med 2015; 30:1349–1358. https://www.ncbi.nlm.nih.gov/pmc/articles/PMC4539313/. Accessed 1 July 2017. 10.1007/s11606-015-3372-9 PMC4539313 26173529

[32] Yevlahova D, Satur J. Models for individual oral health promotion and their effectiveness: a systematic review. Aust Dent J 2009; 54:190–197. http://onlinelibrary.wiley.com/doi/10.1111/j.1834-7819.2009.01118.x/full. Accessed 1 July 2017. 10.1111/j.1834-7819.2009.01118.x 19709105

[33] Enkin M, Keirse JNC, Renfrew M, Nelson J (1995). A guide to effective care in pregnancy and childbirth. Reprod Health Matters.

[34] Jackson N. Handbook for systematic reviews of health promotion and public health interventions. In: The Cochrane Collaboration. 2005. https://ph.cochrane.org/sites/ph.cochrane.org/files/public/uploads/HPPH_systematic_review_handbook.pdf. Accessed 1 July 2017.

[35] Young L, Kent L, Walters L (2011). The John Flynn placement program: evidence for repeated rural exposure for medical students. Aust J Rural Health.

[36] Kitchener S, Day R, Faux D, Hughes M, Koppen B, Manahan D (2015). Longlook: initial outcomes of a longitudinal integrated rural clinical placement program. Aust J Rural Health.

[37] Roberts C, Daly M, Kumar K, Perkins D, Richards D, Garne D (2012). A longitudinal integrated placement and medical students’ intentions to practise rurally. Med Educ.

[38] Jones M, Bushnell J, Humphreys J (2014). Are rural placements positively associated with rural intentions in medical graduates?. Med Educ.

[39] Forster L, Assareh H, Watts L, McLachlan C. Additional years of Australian rural clinical school undergraduate training is associated with rural practice. BMC Med Educ 2013;13: 37. https://www.ncbi.nlm.nih.gov/pmc/articles/PMC3599975/. Accessed 1 July 2017. 10.1186/1472-6920-13-37 PMC3599975 23607311

[40] Eley DS, Synnott R, Baker PG, Chater AB. A decade of Australian rural clinical school graduates--where are they and why?. Rural Remote Health 2012;12:1937. http://www.rrh.org.au/publishedarticles/article_print_1937.pdf. Accessed 1 July 2017. 22394086

[41] Playford D, Evans S, Atkinson D, Auret K, Riley G (2014). Impact of the rural clinical school of Western Australia on work location of medical graduates. Med J Aust.

[42] Isaac V, Watts L, Forster L, McLachlan C. The influence of rural clinical school experiences on medical students’ levels of interest in rural careers. Hum Resour Health 2014;12:48. https://www.ncbi.nlm.nih.gov/pmc/articles/PMC4159525/. Accessed 1 July 2017. 10.1186/1478-4491-12-48 PMC4159525 25169650

[43] Sen Gupta T, Woolley T, Murray R, Hays R, McCloskey T. Positive impacts on rural and regional workforce, from the first seven cohorts of James Cook University medical graduates. Rural Remote Health. 2014;14: 2657. http://www.rrh.org.au/publishedarticles/article_print_2657.pdf. Accessed 1 July 2017. 24645878

[44] Playford D, Cheong E (2012). Rural undergraduate support and coordination, rural clinical school, and rural Australian medical undergraduate scholarship: rural undergraduate initiatives and subsequent rural medical workforce. Aust Health Rev.

[45] Eley D, Baker P (2009). The value of a rural medicine rotation on encouraging students toward a rural career: clear benefits from the RUSC program. Teach Learn Med.

[46] Smedts AM, Lowe MP (2008). Efficiency of clinical training at the Northern Territory clinical school: placement length and rate of return for internship. Med J Aust.

[47] Strasser R, Hogenbirk J, Lewenberg M, Story M, Kevat A (2010). Starting rural, staying rural: how can we strengthen the pathway from rural upbringing to rural practice?. Aust J Rural Health.

[48] Sen Gupta T, Murray R, Hays R, Woolley T. James Cook University MBBS graduate intentions and intern destinations: a comparative study with other Queensland and Australian medical schools.Rural Remote Health. 2013; 13: 2313. http://www.rrh.org.au/publishedarticles/article_print_2313.pdf. Accessed 1 July 2017. 23751066

[49] Eley D, Baker P, Chater B (2009). The rural clinical school tracking project: more IS better – confirming factors that influence early career entry into the rural medical workforce. Med Teach.

[50] Rabinowitz H, Diamond J, Markham F, Rabinowitz C (2005). Long-term retention of graduates from a program to increase the supply of rural family physicians. Acad Med.

[51] Quinn K, Kane K, Stevermer J, Webb W, Porter J, Williamson H (2011). Influencing residency choice and practice location through a longitudinal rural pipeline program. Acad Med.

[52] Kane K, Quinn K, Stevermer J, Porter J, Webb W, Williamson H (2013). Summer in the country: changes in medical students’ perceptions following an innovative rural community experience. Acad Med.

[53] MacDowell M, Glasser M, Hunsaker M (2013). A decade of rural physician workforce outcomes for the Rockford rural medical education (RMED) program University of Illinois. Acad Med.

[54] Rabinowitz H, Diamond J, Markham F, Santana A (2013). Retention of rural family physicians after 20-25 years: outcomes of a comprehensive medical school rural program. J Am Board Fam Med.

[55] Rabinowitz H, Diamond J, Markham F, Santana A (2011). Increasing the supply of rural family physicians: recent outcomes from Jefferson medical Collegeʼs physician shortage area program (PSAP). Acad Med.

[56] Landry M, Schofield A, Bordage R, Bélanger M (2011). Improving the recruitment and retention of doctors by training medical students locally. Med Educ.

[57] Longombe AO. Medical schools in rural areas – necessity or aberration?. Rural Remote Health 2009; 9: 1131. http://www.rrh.org.au/publishedarticles/article_print_1131.pdf. Accessed 1 July 2017. 19653801

[58] Rourke JTB, Incitti F, Rourke LL, Kennard M (2005). Relationship between practice location of Ontario family physicians and their rural background or amount of rural medical education experience. Can J Rural Med.

[59] Jamieson JL, Kernahan J, Calam B, Sivertz KS. One program, multiple training sites: does site of family medicine training influence professional practice location. Rural Remote Health 2013; 13:2496. https://pdfs.semanticscholar.org/fe2a/5d88bb85aaf573fad8ce5e45d84e73bf6685.pdf. Accessed 20 Jan 2018. 24329573

[60] Crump W, Fricker R, Ziegler C, Wiegman D (2015). Increasing the rural physician workforce: a potential role for small rural medical school campuses. J Rural Health.

[61] Smucny J, Beatty P, Grant W, Dennison T, Wolff L (2005). An evaluation of the rural medical education program of the State University of new York upstate Medical University, 1990-2003. Acad Med.

[62] Glasser M, Hunsaker M, Sweet K, MacDowell M, Meurer M (2008). A comprehensive medical education program response to rural primary care needs. Acad Med.

[63] Playford D, Puddey I (2016). Interest in rural clinical school is not enough: participation is necessary to predict an ultimate rural practice location. AJRH.

[64] Pepper C, Sandefer R, Gray M (2010). Recruiting and retaining physicians in very rural areas. J Rural Health.

[65] Chen F, Fordyce M, Andes S, Hart L (2010). Which medical schools produce rural physicians? A 15-year update. Acad Med.

[66] Orzanco MG, Lovato C, Bates J, Slade S, Grand Maison P, Vanasse A, Nature and nurture in the family physician's choice of practice location. Rural Remote Health 2011; 11:1849. https://www.rrh.org.au/journal/article/1849. Accessed 20 Jan 2018. 21919544

[67] Playford D, Ng W, Burkitt T (2015). Creation of a mobile rural workforce following undergraduate longitudinal rural immersion. Med Teach.

[68] Clark T, Freedman S, Croft A, Dalton H, Luscombe G, Brown A (2013). Medical graduates becoming rural doctors: rural background versus extended rural placement. Med J Aust.

[69] Kondalsamy-Chennakesavan S, Eley D, Ranmuthugala G, Chater A, Toombs M, Darshan D (2015). Determinants of rural practice: positive interaction between rural background and rural undergraduate training. Med J Aust.

[70] Walker JH, Dewitt DE, Pallant JF, Cunningham CE. Rural origin plus a rural clinical school placement is a significant predictor of medical students’ intentions to practice rurally: a multi-university study. Rural Remote Health. 2012; 12:1908. http://www.rrh.org.au/articles/subviewnew.asp?ArticleID=1908. Accessed 1 July 2017. 22239835

[71] Herd M, Bulsara M, Jones M, Mak D (2016). Preferred practice location at medical school commencement strongly determines graduates' rural preferences and work locations. Aust J Rural Health.

[72] Wendling A, Phillips J, Short W, Fahey C, Mavis B (2016). Thirty years training rural physicians. Acad Med.

[73] Worley P, Martin A, Prideaux D, Woodman R, Worley E, Lowe M (2008). Vocational career paths of graduate entry medical students at Flinders University: a comparison of rural, remote and tertiary tracks. Med J Aust.

[74] Pagaiya N, Kongkam L, Sriratana S. Rural retention of doctors graduating from the rural medical education project to increase rural doctors in Thailand: a cohort study. Hum Resour Health 2015;13:10. https://human-resources-health.biomedcentral.com/articles/10.1186/s12960-015-0001-y. Accessed 1 July 2017. 10.1186/s12960-015-0001-y PMC4355566 25889590

[75] Rabinowitz H, Petterson S, Boulger J, Hunsaker M, Diamond J, Markham F (2012). Medical school rural programs: a comparison with international medical graduate in addressing state-level rural family physician and primary care supply. Acad Med.

[76] Zink T, Center B, Finstad D, Boulger J, Repesh L, Westra R (2010). Efforts to graduate more primary care physicians and physicians who will practice in rural areas: Examining outcomes from the University of Minnesota–Duluth and the Rural Physician Associate Program. Acad Med.

[77] Brokaw J, Mandzuk C, Wade M, Deal D, Johnson M, White G et al. The influence of regional basic science campuses on medical students' choice of specialty and practice location: a historical cohort study. BMC Med Educ 2009; 9:29. https://bmcmededuc.biomedcentral.com/track/pdf/10.1186/1472-6920-9-29?site=bmcmededuc.biomedcentral.com. Accessed 20 Jan 2018. 10.1186/1472-6920-9-29 PMC2700105 19500392

[78] Playford D, Nicholson A, Riley G, Puddey I. Longitudinal rural clerkships: increased likelihood of more remote rural medical practice following graduation. BMC Med Educ. 2015;15(1). https://bmcmededuc.biomedcentral.com/track/pdf/10.1186/s12909-015-0332-3?site=bmcmededuc.biomedcentral.com. Accessed 20 Jan 2018. 10.1186/s12909-015-0332-3 PMC4372318 25879715

[79] Kwan M, Kondalsamy-Chennakesavan S, Ranmuthugala G, Toombs M, Nicholson G (2017). The rural pipeline to longer-term rural practice: general practitioners and specialists. PLoS One.

[80] Wright J, Bourke L, Waite C, Holden T, Goodwin J, Marmo A (2014). A short-term rural placement can change metropolitan medical students' knowledge of, and attitudes to, rural practice. Med J Aust.

[81] Somers G, Spencer R (2012). Nature or nurture: the effect of undergraduate rural clinical rotations on pre-existent rural career choice likelihood as measured by the SOMERS index. Aust J Rural Health.

[82] Matthews C, Bagg W, Yielder J, Mogol V, Poole P. Does Pukawakawa (the regional-rural programme at the University of Auckland) influence workforce choice?. N Z Med J 2015;128:35–43. https://www.nzma.org.nz/journal/read-the-journal/all-issues/2010-2019/2015/vol-128-no-1409/6437. Accessed 1 July 2017. 25721960

[83] Myhre DL, Woloschuk W (2016). Practice locations of longitudinal integrated clerkship graduates: a matched-cohort study. Can J Rural Med.

[84] Stagg P, Greenhill J, Worley PS. A new model to understand the career choice and practice location decisions of medical graduates. Rural Remote Health 2009;9:1245. http://www.rrh.org.au/publishedarticles/article_print_1245.pdf. Accessed 1 July 2017. 19943714

[85] Eley DS, Baker PG (2007). Will Australian rural clinical schools be an effective workforce strategy? Early indications of their positive effect on intern choice and rural career interest. Med J Aust.

[86] Jamar E, Newbury J, Mills D. Early career location of University of Adelaide rural cohort medical students. Rural Remote Health. 2014;14: 2592. http://www.rrh.org.au/publishedarticles/article_print_2592.pdf. Accessed 1 July 2017. 24506734

[87] Eley D, Baker P, Does recruitment lead to retention? Rural Clinical School training experiences and subsequent intern choices Rural Remote Health 2006;6:511. http://www.rrh.org.au/publishedarticles/article_print_511.pdf. Accessed 1 July 2017. 19469660

[88] Lee YH, Barnard A, Owen C, Initial evaluation of rural programs at the Australian National University: understanding the effects of rural programs on intentions for rural and remote medical practice. Rural Remote Health. 2011;11: 1602. http://www.rrh.org.au/publishedarticles/article_print_1602.pdf. Accessed 1 July 2017. 21568620

[89] Birden HH, Wilson I. Rural placements are effective for teaching medicine in Australia: evaluation of a cohort of students studying in rural placements. Rural Remote Health 2012;12:2167. http://www.rrh.org.au/articles/subviewnew.asp?ArticleID=2167. Accessed 1 July 2017. 23157496

[90] Gerber J, Landau L (2010). Driving change in rural workforce planning: the medical schools outcomes database. Aust J Rural Health.

[91] Williamson MI, Wilson R, McKechnie R, Ross J. Does the positive influence of an undergraduate rural placement persist into postgraduate years?. Rural Remote Health 2012; 12: 2011. http://www.rrh.org.au/publishedarticles/article_print_2011.pdf. Accessed 1 July 2017. 22713111

[92] Denz-Penhey H, Shannon S, Murdoch CJ, Newbury JW. Do benefits accrue from longer rotations for students in rural clinical schools? Rural Remote Health 2005; 5:414. http://www.rrh.org.au/publishedarticles/article_print_414.pdf. Accessed 1 July 2017. 15946108

[93] Deveney K, Deatherage M, Oehling D, Hunter J (2013). Association between dedicated rural training year and the likelihood of becoming a general surgeon in a small town. JAMA Surgery.

[94] Greer T, Kost A, Evans D, Norris T, Erickson J, McCarthy J (2016). The WWAMI targeted rural underserved track (TRUST) program. Acad Med.

[95] Petrany S, Gress T (2013). Comparison of academic and practice outcomes of rural and traditional track graduates of a family medicine residency program. Acad Med.

[96] Crampton P, McLachlan J, Illing J (2013). A systematic literature review of undergraduate clinical placements in underserved areas. Med Educ.

[97] Victora C, Habicht J, Bryce J. Evidence-based public health: moving beyond randomized trials. Am J Public Health 2004; 94:400–405. https://www.ncbi.nlm.nih.gov/pmc/articles/PMC1448265/. Accessed 1 July 2017. 10.2105/ajph.94.3.400 PMC1448265 14998803

[98] Farmer J, Kenny A, McKinstry C, Huysmans RD (2015). A scoping review of the association between rural medical education and rural practice location. Hum Resour Health.

[99] Barrett F, Lipsky M, Lutfiyya NM (2011). The impact of rural training experiences on medical students: a critical review. Acad Med.

[100] O’Sullivan B, McGrail M, Russell D, Chambers H, Major L (2018). A review of characteristics and outcomes of Australia’s undergraduate medical education rural immersion programs. Hum Resour Health.

[101] Humphreys JS, Prideaux D, Beilby JJ, Glasgow NJ (2009). From medical school to medical practice: a national tracking system to underpin planning for a sustainable medical workforce in Australasia. Med J Aust.

[102] Mason J. Review of Australian government health workforce programs. Canberra: Department of Health and Ageing, 2013. file:///C:/Users/40051052/AppData/Local/Microsoft/Windows/INetCache/IE/P2YO3WZJ/Review%20of%20Health%20Workforce%20programs.pdf. Accessed 20 June 2018.

[103] Wilson N, Couper ID, Vries E, Reid S, Fish T, Marais BJ (2009). A critical review of interventions to redress the inequitable distribution of healthcare. Rural Remote Health.

[104] Smedts MA, Lowe MP. Clinical training in the top end: impact of the northern territory clinical school, Australia on the territory’s health workforce Rural Remote Health 2007; 7:723. http://www.rrh.org.au/publishedarticles/article_print_723.pdf. Accessed 1 July 2017. 17489646

[105] Rabinowitz H, Diamond J, Markham F, Wortman J (2008). Medical school programs to increase the rural physician supply: a systematic review and projected impact of widespread replication. Acad Med.

[106] Rabinowitz H, Diamond J, Markham F, Hazelwood C (1999). A program to increase the number of family physicians in rural and underserved areas. JAMA.

[107] Henry JA, Edwards BJ, Crotty B. Why do medical graduates choose rural careers?. Rural Remote Health 2009; 9:1083. http://www.rrh.org.au/publishedarticles/article_print_1083.pdf. Accessed 1 July 2017. 19257797

[108] Department of Health. In: Review of Australian Government Health Workforce Programs. 2013. http://www.health.gov.au/internet/publications/publishing.nsf/Content/work-review-australian-government-health-workforce-programs-toc. Accessed 1 July 2017.

[109] Playford D, Ngo H, Gupta S, Puddey I (2017). Opting for rural practice: the influence of medical student origin, intention and immersion experience. The MJA.

