# Supplementary material for: The impact of rural outreach programs on medical students’ future rural intentions and working locations: a systematic review
Source: BMC Med Educ. 2018 Aug 14;18:196. doi: 10.1186/s12909-018-1287-y (PMC6092777; doi:10.1186/s12909-018-1287-y)
Supplement: Supplementary file 1 — Appendix S1. A summary of the 62 selected studies. Provides the reader with summary information of the 62 selected studies specific characteristics and outcomes. (DOCX 77 kb) [file 12909_2018_1287_MOESM1_ESM.docx]

**Additional file 1 – A summary of the 62 selected studies**

| **Author** | **Ref No.** | **Study Location** | **Study Purpose/Aim** | **Study Design** | **Study Population** | **Outcome Measures** | **Results/Significance** |
| --- | --- | --- | --- | --- | --- | --- | --- |
| Critchley J et al. 2007 | 12 | Australia | Report on student responses to a 4-week rural health module and rural placements. | Descriptive Study | 368 students participated out of 393. | Pre and Post  Quantitative and Qualitative survey questions | The rural health course positively influenced students’ intention for rural work and increased students interest in rural health.  Study noted that most participants are from an urban background. |
| Young L et al. 2011 | 35 | Australia | Reports on a rural placement program which provides a rural and medical experience, to increase rural career intentions. | Longitudinal Descriptive tracking study | 688 students | Post only and annual follow up | 85% of students reported their participation in the program increased students’ rural ambitions.  This initial longitudinal data indicates the program is having a positive impact on the recruitment of rural doctors. |
| Kitchener S et al. 2015 | 36 | Australia | Study looks at the academic performance and career choices of students who participated in a longitudinal integrated rural placement. | Retrospective, observational, cohort  study | 683 graduates | Early career choices were obtained for graduates  employed by Queensland Health through the  Queensland Intern Matching Process. | 67% of participants have undertaken an internship at a rural location, compared with 15% of non-participants who attended urban hospital-based placements. Study indicates positive outcomes in terms of encouraging graduates to find work in rural settings. |
| Roberts C et al. 2012 | 37 | Australia | Looked at the effect of an integrated placement on students’ attitudes towards rural employment | Qualitative Study applying Socio-Cognitive Theory | Interviews with 10 medical students, 15 clinical supervisors and teachers, 3 community health staff, and focus groups. | Pre-and post-placement interviews. | This longitudinal placement program with an informal curriculum positively influenced students rural work intentions. |
| Jones M et al. 2014 | 38 | Australia | To determine if rural clinical placements are associated with a higher proportion of graduates’ rural intentions | Longitudinal retrospective | 3268 graduates | Entry and Exit questionnaire  Medical  Schools Outcomes Database  Study  prospectively  collects data, including practice location  intention, from all Australian medical schools. | Remote placements were associated with a 77% increase in the odds of intention to become a rural doctor. The factors that positively influence students’ rural intentions are; exposure to rural practice during training, location and curriculum focus of a medical school.  Rural origin and early intentions at the start of their medical training are stronger predictors of rural intentions than rural clinical placements. |
| Forster L 2013 | 39 | Australia | Evaluate the impact of an Australian rural clinical school (RCS) on graduate current, preferred current and intended location for rural employment. | Retrospective longitudinal survey | Yes (214/315) graduates between the years 2003– 2010 | Post online survey | Beyond 1 year at an RCS for undergrad medical students is associated with current work location, preferred current work location and intended work location in a rural area.  3 years in an RCS significantly increases the likelihood of rural career intentions of non-rural students. |
| Eley DS et al. 2012 | 40 | Australia | RCS Study looking at the impact of a rural undergraduate clinical training program on the workforce choices of graduates, who have been graduated for up to 9 years. | Longitudinal mixed methods, sequential explanatory design. | 115/180 of graduates | Online questionnaire and semi-structured interviews - data merging.  Tracking database established in 2006, in 2010, all former grads emailed a questionnaire.  29 interviewed (64%). | 40% of respondents were currently working in non-urban locations.  Reported strong positive influence that rural clinical training increases rural intentions. However, study notes that these intentions are impacted by concurrent personal/life choices.  Study reported the longer the exposure to rural training, the greater the impact on rural intentions. |
| Playford DE  et al. 2014 | 41 | Australia | Impact of a year in a rural clinical school of Western Australia (RCSWA) on influencing graduates work enter the rural workforce  10 year follow up study. | Multiple Cohort Cross sectional study | 1017 graduates | Used AHPRA to track graduates | Of 258 RCSWA graduates, 16.3% were working rurally compared with 4.7% of the control group. Undertaking the RCSWA program is strongly associated with increased likelihood of working rurally.  Urban background grads who took part in the RCSWA were much more likely to work rurally than those who did not. |
| Isaac et al. 2014 | 42 | Australia | Looking at the impact of one year at a RCS on self-reported interest in a rural career and career intentions. Also, looking at the factors that relate to rural interest/intent. | Descriptive study and short term follow up. | 150 students | Self-administered UNSW undergrad questionnaire.  Data collected at baseline and after one year of RCS training on preferred  location for internship, work and intended specialty. | Factors associated with intent to practise in a rural location were rural background, 2 or  more previous years at an RCS, and preference for a rural internship.  At follow-up 41% of participants who originally intended to work in a metropolitan location at baseline changed their preference and indicated a preference for a rural location.  An RCS experience can increase rural work intent and levels of interest in rural practice. |
| Sen Gupta T et al. 2014 | 43 | Australia | Shows working location evidence for first seven cohorts of graduates from new RCSs. Study also aims to show associations between applicants’ origin, internship location and practice location. | Longitudinal Cohort Study | 530 graduates | Contact was made by email and telephone communication, and via a JCU School of  Medicine Facebook page, which was initiated on student  advice in 2005 and has been continued since for all graduating  cohorts.  Follow up data using AHPRA and other medical databases. | Study reports positive trends of the seven cohorts toward leading to significant workforce increase in northern and or rural Australia. |
| Playford &  Cheong 2012 | 44 | Australia | Followed up the working locations of graduates who spent an undergraduate clinical year spend in a Rural Clinical School of Western Australia (RCSWA).  Used a 6-week Rural Undergraduate Support and Coordination (RUSC) course as a comparison group. | Retrospective longitudinal cohort study | 682 graduates | Post follow up of graduates working locations  2 cohorts:  One year spent in an undergraduate rural clinical school  6 weeks in a rural under support and coordination experience out of a 6-year med degree | RCSWA was associated with significantly more postgraduate year one work rural work than the RUSC placement alone.  The RCSWA workforce effect increased at postgraduate year two of the program.  Rural-origin practitioners who chose the RCSWA program were more likely than other rural-origin practitioners to take rural rotations in both postgraduate years. |
| Eley D and Baker P 2009 | 45 | Australia | This article reports on the impact of an Australian Rural Clinical  School in terms of if it encourages students to adopt a rural career. | Descriptive study | 463 students | Pre-and post-survey | A rural rotation can encourage students’ interest in and understanding of rural medicine |
| Smedts AM & Lowe MP 2008 | 46 | Australia | To investigate the impact of duration of clinical training placements in the Northern Territory on rate of return of medical students for an internship in the NT. | Retrospective survey | 683 students  The total number of weeks spent rural training was ascertained from student and admin records. | Alumni and hospital records ascertained the total number of weeks of rural training. | An increased exposure to the rural clinical training in the Northern Territory led to an increased rate of return. The study identified that short term placements in the final year, yielded the least return for the number of weeks of placement provided. |
| Strasser et al. 2010 | 47 | Australia | The program looked at four groups at Monash University to assess if after the completion of rural clinical training, did rural background affect the respondents intended place of employment. | Retrospective cohort study | All rural background students enrolled in the Monash MBBS program between 1992 and 1999 and to 25-56% of urban background students from each year.  83 completed questionnaires. | Retrospective cohort mail survey. Pre-and Post-Placement Questionnaires | Study reported that increased interest in rural practice in the early postgraduate years for both rural background and non-rural background is not reflected in actual practice location after graduation.  Evidence did show that the RUSC initiatives were increasing intent for rural practice. Longer rural placements were associated with increased rural intent and a preferred rural practice location in 5-10 years’ time. |
| Sen Gupta et al. 2013 | 48 | Australia | James Cook University (JCU) School of Medicine. Program was created to address the rural/urban divide in the medical workforce through aligning student selection, curriculum and assessment practices (rural clinical placements – 20 weeks) to encourage generalist in rural locations. The study reports on the early outcomes of graduates in the first 6 cohorts from 2005 to 2010. | Retrospective Survey | A total of 292 Year 6 JCU MBBS students out of a possible  445 completed the exit survey from 2005 to 2010, an overall  response rate of 66%.  This information was compared with other Queensland MSOD data (720 participants from other schools and 1457 participants from 6 other Universities in different states. | Post survey and MSOD data. | The majority of JCU medical students intended to work outside of the capital cities compared with 31% from the MSOD (other Unis) data. |
| Eley D et al. 2009 | 49 | Australia | Looks at the early career pathways of alumni from a RCS, and reports on the perceptions of the rural school on rural intentions and the factors that influences these choices. | Longitudinal retrospective cohort study | 180 participants tracked, with a 69% response rate. | Survey collected the following information; contact details (email addresses), demographic questions, education, rural background, rural experience, and current job title identified. | Found that longer rural clinical experience is more effective in the working location choice and future interest in working rurally. |
| Rabinowitz, et al. 2005 | 50 | United States | Study of the long-term retention of  graduates of the Physician Shortage Area Program (PSAP) of Jefferson Medical College. | Longitudinal tracking project | 92 graduates | Tracked in 2002 and went back 30 years.  Applied previous pre-and post-questionnaire data. | After 11–16 years, 68%) of the PSAP graduates were still practicing family medicine in the same rural area, compared with 46% of their non-PSAP peers.  Study shows long term rural primary care retention rates and the rural program (PSAP) participants has led to both increases in recruitment (8-fold) and long-term retention. |
| Quinn K et al. 2011 | 51 | United States | Report on specialty choices and first working locations of the graduates of the three intervention cohorts. Rural Program known as the Missouri School Rural Track Pipeline Program (MU-RTPP) and consisted of 3 types of rural cohorts. | Longitudinal Multiple intervention tracking study | 48 Rural Scholars –  83 6month Rural Track Clerkship (RTC) for 3rd year students  51, Rural Track Elective Program for 4th year students  506 nonparticipants | Compared residency selection choices using medical and workforce databases | Rural Scholars were more than twice as likely to match into family medicine.  RTC participants entered primary care, especially family medicine, at rates significantly higher than nonparticipants.  Over 57% of students who participated in the RTC program (and potentially other MU-RTPP offerings) chose a rural location for their first practice. |
| Kane K et al. 2013 | 52 | United States | Reviewing a summer community rural program aimed at increasing students’ interest in rural practice. | Longitudinal (descriptive study) pipeline program | 229 students | Pre-and post-questionnaires  Used tracking databases | 72% reported increased interest in rural practice.  46% of participants decided to work in rural areas for their first practices  Compared with the control group, summer rural participants were more likely to enter a primary care residency and twice as likely to choose specifically family medicine |
| MacDowell M. et al. 2013 | 53 | United States | To report on the working locations of graduates from the University of Illinois College of Medicine at Rockford Rural Medical Education Program (RMED) | Longitudinal tracking project | 160 RMED  2663 NON-RMED | Follow up working locations using medical and workforce databases | RMED graduates were 14.4 times more likely than non-RMED graduates to choose family medicine; 6.7 times more likely to choose a primary care practice specialty and 17.2 times more likely to be currently practicing in a rural location. |
| Rabinowitz H et al. 2013 | 54 | United States | Applied the Jefferson Longitudinal Study to compare the numbers of PSAP and non-PSAP in 2011 who were still working in family medicine in the same rural location (looked retention).  A look to see if certain graduates were still working rurally 25 years later in family medicine. | Longitudinal tracking project | 90 graduates from the JMC  37 PSAP  52 non -PSAP | Using the Jefferson Longitudinal study, this study compared the numbers of PSAP and non-PSAP who were still working in rural family medicine in 2011.  Study used databases (American Board of Medical Specialties and self-reported specialty data from the AMA Physician Masterfile.  . | The study provides further evidence that graduates of rural programs are likely to enter rural family medicine and that these graduates will remain in these practices for decades. |
| Rabinowitz H et al. 2011 | 55 | United States | Looked at the geographic and speciality work choices of graduates from a longitudinal PSAP rural placement program. | Longitudinal Study | 104 PSAP graduates (intervention)  2281 non-PSAP (control) | Used established databases – (AMA Physician Masterfile), JLS database, American Board of Medical Specialties and merged the data with the alumni information. | PSAP graduates were 10 times more likely to be working rural family medicine than non-PSAP cohort. |
| Landry et al. 2011 | 56 | Canada | Researched the effects of frequency and length of rural province exposure during medical training on the likelihood of rural intentions or actual employment in that same province. | Cross-sectional survey | 260/390 | Questionnaire via phone interview or in writing. | Study reported that the length, timing and frequency of exposure to the regional area during medical training was positively associated with the likelihood that a doctor will go onto work in that same region. |
| Longombe AO 2009 | 57 | Africa | The initial outcomes of a rural located medical school in the DRC are reported, and compared with an urban medical school | Longitudinal follow up study | 43 Rural UNI  and 107 Metro UNI  . | Alumni records were used.  Looked at rural or urban employment via alumni records.  . | Results support the establishment of rural medical schools (in rural areas), with the majority of rural school participants working rurally after graduation.  Study therefore provides evidence that rural located medical schools can increase the numbers of physicians working in rural areas in the DRC. |
| Rourke JTB 2005 | 58 | Canada | Study designed to determine if there was a difference in rural background and rural medical education experience between practicing rural physicians and practising urban physicians in Ontario. | Cross sectional study | 507 rural family physicians and 505 urban family physicians in Ontario  264 rural responded  179 urban responded. | Main outcome measure was population of the community while growing up, rural medical education and medical school attended. | The study reported that the rural physicians were significantly more likely to have grown up in a rural community, to have received rural clinical training during medical school, to have graduated outside of the major city of Ontario, and to have had 8 weeks or more rural training during their postgrad residency training. |
| Jamieson J et al. 2013 | 59 | Canada | Practice location of family physicians who participated in a postgraduate training based at either metropolitan or distributed non-metropolitan communities. | Longitudinal retrospective tracking study | 45% response rate. | Key outcome was workforce location, rurality of location and preparedness for rural practice.  Questionnaire survey mailed to graduates. Sent at 2 years, 5 and 10 years after completing the postgrad training.  Master file of all graduates, is maintained by the Department of Family Practice postgraduate program. | Resident trained in the distributed non-metropolitan sites were 15 times more likely to enter rural practice, small town and regional centres than the metropolitan trained residents.  The positive impact of distributed postgraduate family medicine training is an important predictor of non-metropolitan practice, and the effect persists for 10 years upon completion of training. |
| Crump WJ et al. 2015 | 60 | United States | A study of a rural immersion experience (rural campus added to the school), looking at the effects of rural upbringing and rural training on choice of practice location. | Retrospective Longitudinal Study | The database constituted 1,120 medical school graduates from 2001 to 2008  Looked at those who participated in the immersion experience and those that did not. | The impact of adding a rural clinical campus to the school on workforce outcomes. In addition, analysed rural upbringing, demographics, family medicine, campus participation and residency choice.  The American Medical Association (AMA) Physician Master File was used to determine location of practice. | Two years in the rural clinical campus showed the strongest association of all the variables with rural practice. Rural upbringing, family medicine residency with rural practice choice all reported positive associations with rural practice. |
| Smucny J et al. 2005 | 61 | United States | Study aim is to assess the success of the program in providing a valuable educational experience and looking at their workforce outcomes/geographic distribution of the graduates.  . | Longitudinal tracking project and retrospective study | Questionnaires were given to 132 RMED graduates about the importance of RMED in their subsequent choices of practice locations.  76 RMED graduates (58%) completed the questionnaire. | - Geographic distribution of the program graduates compared against the non-graduates was identified through the AMA Masterfile. - A look at the importance of the program in the choice of the graduates working location through a voluntary Questionnaire mailed out to the graduates. - A look at the academic performance of the students and managements views. | Approximately four times the RMED graduates were working in a rural clinical practice compared to the control group who did not participate in the RMED.  The RMED program provided a valuable educational experience and assisted the recruitment of rural physicians. |
| Glasser M et al.  2008 | 62 | United States | The study presents the characteristics of the Rural Medical Education (RMED) Program, which implements a system of recruiting students from rural backgrounds and provides a rural focused curriculum. Includes a 16-week rural preceptorship (working with primary care clinicians) within the curriculum.  The study tracks the outcomes via the working locations of the graduates. | Longitudinal tracking project | Since 1993, 216 students have been enrolled in the program.  RMED and non-RED groups. | Workforce outcomes of the program graduates.  MCAT educational scores to assess differences between the RMED students and non-RMED students. | 159 of the 216 have graduated, with 64.4% working in primary care in small towns and/or rural communities.  MCAT scores were similar for RMED and non-RMED students.  The study comments the program compares well with other studies in terms of its positive rural workforce association. |
| Playford D & Puddey IB 2016 | 63 | Australia | A look at the impact of rural exposure during medical school (1 academic year in a rural location in the penultimate year of their course) on workforce outcomes, and specifically how much is due to pre-existing interest in rural employment and how much is related to the rural placements. | Cohort Study | All 1026 University of Western Australia students who had an opportunity to apply for a year-long rotation in RCSWA from 2004 to 2010, and who had subsequently graduated by the end of 2011, were included.  Three cohort groups consisted of;   1. Those who did not apply for a place in the Rural Clinical School Western Australia (RCSWA) 2. Those who applied for a place in RCSWA, but did not get in. 3. Those who applied and got in to the RCSWA | Graduates’ principal work- place location using AHPRA. | RCSWA graduates were three times more likely to work in a rural location compared to either control group. The study suggests the RCSWA has a significant independent effect on rural workforce. |
| Pepper CM et al. 2010 | 64 | United States | The study looked at the variables associated with rural background and training, to identify if they predicted clinicians rural workforce outcomes | Longitudinal retrospective tracking project | 1,218 physicians initially identified, 996 confirmed that they were practicing in Wyoming. Of those, 693 (69.6%) physicians completed the survey  . | Questionnaire collected their background, current practice, and future practice plans. | A rural background and training in nearby states did predict working in a very rural location.  A predictor identified was malpractice insurance rates, which encourage clinicians to move out of state, rather than within state. |
| Chen F et al. 2010 | 65 | United States | A large longitudinal tracking study of the training of rural physicians in the United States. | Longitudinal tracking study | 175,649 who graduated between 1988 to 1997 and were clinically active physicians were followed up. | Using the AMA database and the American Osteopathic Association Masterfile physician data clinically active physicians were identified, | 11% (20,037) of the cohort were practicing in a rural location in 2005. 18% (2045) of osteopathic medical school graduates were practicing in a rural area. Rural residency trainees were more than 3 times likely to work in a rural location than non-rural residency trainees. |
| Orzanco C et al. 2011 | 66 | Canada | The study looked at two medical education programs in Canada. It focussed on medical student characteristics (nature) and the effect of a rural program (nurture) as predictors of family practice in a non-metropolitan location. Specifically, the study aimed to explore the factors that predict the graduates working location, 2 years post residency training. | Retrospective analysis study  . | All students who registered in the undergrad medical programs and studies family medicine between 1995 and 2000. This lead to Uni 1= 780 and Uni 2 = 936.  Mandatory rural internship - min of 4 weeks for intervention. | Retrospective analysis of two medical education programs. Databases were developed from alumni records.  Working location was the key outcome. | Rural background was the key nature predictor, and the key nurture variable was the length of the non-metropolitan internship.    The third-year clerkship in a rural area may increase the chances of non-metropolitan practice. |
| Playford D et al. 2015 | 67 | Australia | The study is within The Rural Clinical School of Western Australia (RCSWA) and looked at the workforce choices of graduates up to 10 years from a longitudinal immersion rural programme.  Intervention involved one academic year in a rural location as a medical student. | Longitudinal cohort study | 417 consenting graduates.  For this longitudinal cohort study, all consenting graduates were contacted annually after graduation, with the outcome measure being rural work location (defined by the Australian Standard Geographical Classification –Remoteness Area) of any duration. | Survey was emailed with the key outcome measure being rural work location. | The study reported that there was considerable movement in and out of rural employment and the rural workforce was far more mobile than anticipated. However, there were positive associations with rural work from the participants. One year was suggested to be a suitable amount of time to convert commitment to rural work. |
| Clark T et al. 2013 | 68 | Australia | To determine if extended rural placements are associated with intentions to complete rural internships  Rural Placement extended, compared against rural internship and finally rural background | Longitudinal cohort study | Follow up of 3 cohorts – 448 students  98/448 students did the extended rural placement aspect – 32-week rural placement.  35/434 did a rural internship  The last cohort were students from a rural background | Self-reported questionnaires pre-and post-entry to medical school | Increased intentions to undertake rural internships and choosing rural internships after completing medical school.  For all the 3 cohorts, extended placements in rural clinical schools led to a stronger association than rural background with a preference and acceptance for rural internships |
| Kondalsamy- Chennakesavan S et al. 2015 | 69 | Australia | To ascertain the impact of rural background and years of rural clinical school training on graduate workforce outcomes.  . | Retrospective Cohort Study | University of Queensland (UQ) medical graduates who graduated during the period 2002–2011. 1572 graduates were identified and 758 (48%) completed the survey questionnaire.  Two cohorts, the RCS (276) and the metropolitan school attendees (478). | Current clinical practice in a rural location. Location identified via internet, phone, AHPRA, and a questionnaire. | Of the RCS attendees, 41.7% were working rurally, compared to 18.8% working rurally, of the metropolitan school attendees.  Independent predictors of rural practice were identified as; one year in a RCS, 2 years in an RCS, rural background, being single and having a bonded scholarship.  The study identified a number of predictors of rural employment, with a strong interaction identified between rural background and RCS exposure. |
| Walker JH et al. 2012 | 70 | Australia | Study aims to identify the factors that affecting rural practice of students completing a RCS program.  . | Descriptive study | 125 students | Questionnaires post rural placement | Students from rural backgrounds were 10 times more likely to prefer to work in rural areas when compared with other students (p<0.001)  85% of students reported that their RCS experience increased their interest in rural training and practice.  62% indicated a preference for rural internship/basic  training after their RCS experience. Student rural background was reported as the most significant predictor of rural intention. |
| Herd et al. 2016 | 71 | Australia | To identify the factors influencing whether Australian medical graduates prefer to work rurally or go onto work in rural locations | Longitudinal tracking project and retrospective study | 4028 out of 20784 - completed the pre and post questionnaire - (20784 completed the pre and 4028 completed both - post was 1 and 3 years post grad). | Preferred and actual employment locations at one and three years’ post-graduation. | Self-reported preferred practice location at medical school commencement was the most consistent predictor of preferred rural practice location and completing a rural rotation in the first and third years after graduation.  Preferred practice location at medical school commencement is independent of, and enhances the effect of, rural background |
| Wendlin AL et al. 2016 | 72 | United States | The Rural Physician Pipeline was developed in 1974 by the Michigan State University College of Human Medicine (MSU-CHM).  The study presents 30 years of workforce outcomes of the program participants, and the patterns of specialty choices.  . | Retrospective Longitudinal Study | 179 students graduated from the RPP between 1978 and 2006, and 168 (94%) identified.  2610 graduated from the other school campuses and were included and identified (93%) (Control).  . | AMA Masterfile allowed for cross-sectional analysis of all the graduates within the period. Speciality choice, practice location, and graduate outcomes all key outcomes compared over time.  Demographic information was obtained from the American Medical College Application Service database. | RPP graduates were more likely to practice primary care, practice a high-risk specialty, practice rurally, and an area with a health professional shortage.  The study identified that programs should target rurally interest students, provide significant clinical training in a rural area and this can lead to physicians working rurally. |
| Worley P et al. 2008 | 73 | Australia | To follow up the working locations of medical graduates from the remote workforce program at Flinders University (the Parallel Rural Community Curriculum (PRCC) and the Northern Territory Clinical School (NTCC), and comparing them with students at the urban Flinders Medical Centre (FMC) | Retrospective Study | 74/150 | Retrospective postal survey of graduates. | The two rural programs increased the likelihood of graduates choosing a rural career (even when accounting for age and rural background). |
| Pagaiya et al. 2015 | 74 | Thailand | The rural medical education programme ‘Collaborative Project to Increase Rural Doctors (CPIRD) is designed to attract and retain rural doctors. This study assessed the impact of the CPIRD in relation to doctor retention in rural areas and public health service, with an aim to increase the number of rural doctors through increased medical opportunities for students with a rural background. | Cross sectional longitudinal study | 7,157 doctors  who graduated and joined the MoH service between  2000 and 2007. Of these, 1,093 graduated from the  CPIRD track and 6,064 from the normal track.  100% follow up rates. | Baseline data collected, and follow-up data and workplace location were, number of years in rural districts and years within the MoH service.  Data collected from 2000 to 2011 and  Follow-up rates were 100% for the entire  study population. | The study reported a positive impact as CPIRD doctors were more likely to stay working in rural areas and in public service than their peers who did not complete the rural training program. |
| Rabinowitz HK et al. 2012 | 75 | United States | Looks at rural primary care practice outcomes for rural placement graduates compared with international medical graduates (IMGs). | Longitudinal tracking study | Graduates  2 Cohorts  1757 graduates from 3 rural placements  6574 IMGs | Follow up of graduates working locations using data from the 2010 American  Medical Association Physician Masterfile, | Rural placement graduates were 10 times more likely to practice rural family medicine than IMGs and almost 4 times as likely to practice any rural primary care specialty.  The rural placements significantly increased work in rural family physician and primary care work compared to the IMGs. |
| Zink T et al. 2010 | 76 | United States | To compare  Medical education programs at the University of Minnesota (UMN) aimed at increasing the number of rural and primary care physicians  Key rural program was a 9 month RPAP immersion experience | Longitudinal tracking study | 3365 is entire sample  Three interventions and one control | All surveys and newsletters applied to Graduates in addition to internal and external databases. Internet and web searches were to ascertain the working practice location of graduates when information was incomplete. | The RPAP experience led to the highest number of rural primary care physicians.  The study reported that admitting students with an interest in rural primary care and exposing them to rural experiences via a longitudinal immersion experience (RPAP) can lead to metropolitan raised students working in rural practice.  Both programs (RPAP) and UMN-Duluth led to more graduates working rurally  . |
| Brokaw et al. 2009 | 77 | United States | Study followed up the working locations of 2487 physicians who graduated in the decade 1988-1997 to assess the impact on working locations of a 2-year training in a rural location. | Longitudinal tracking study | Followed up 2487 graduates | Alumni records were matched with data from the 2003 AMA Physician Masterfile to ascertain the self-reported medical specialty and working location of the graduates. | Findings suggest that a regional campus environment during the first 2 years of medical school can encourage students to consider regional career paths.  Attending a regional campus was a significant predictor of both medical speciality choice and practice location. |
| Playford D et al.  2015 | 78 | Australia | The study acknowledges that extended rural clerkships lead to an increased likelihood for rural employment. The study is focussed on if these clerkships lead to graduates working in more remote areas, as opposed to regional locations.  . | Retrospective longitudinal cohort study | 3282 graduates of the University of Western Australia Medical School who entered from 1980 and had completed the course by 2011, 3020 were able to be tracked.  . | In May 2014, the Australian Health Practitioner Regulation Agency (AHPRA) database was used to identify the current workplace of every graduate of the Medical School of Western Australia.  3 cohort groups (2 control groups). | Of the intervention group, 78.7% were practicing in outer regional/very remote locations. Those not participating in the RCSWA (2 groups) had 45.5% and 52.4% working in outer regional/very remote locations.  Significant predictors of working in more remote rural practice, were being female, and participating in the intervention (The RCSWA).  The intervention (an extended rural clinical clerkship) during medical undergraduate, led to much greater likelihood of practicing in more remote, under-serviced rural locations. |
| Kwan M et al. 2017 | 79 | Australia | The workforce impact of an Australian Rural Clinical School and the predictors of graduates longer term rural practice, and if these predictors differ between general practitioners and specialists. | A cross-sectional cohort study, | 729 medical graduates of The University of Queensland from 2002–2011. | Graduates practice location and primary place of practice (categorised as rural for at least 50% of time since graduation), known as Longer Term Rural Practice (LTRP). Looked at this outcome for both GPs and medical specialists.  Exposures were background, attendance at a metropolitan clinical school or at a rural clinical school for 1 academic year or 2 years. | Independent predictors of LTRP were; rural background, RCS for 1 year, RCS for 2 years, being a GP, and a bonded scholarship.  Specialists were less likely than GP to in an LTRP; however they still exhibited similar positive associations to the predictors identified.  Study advised both GPS and specialists can be encouraged to work rurally, by enrolling rural background medical students and providing them with a long term rural clinical undergraduate training. |
| Julian R Wright et al. 2014 | 80 | Australia | Measure the effect of a short-term placement on metropolitan students’ attitudes toward and knowledge of rural health issues. | Descriptive study | 69 students | Pre-and post-questionnaire  Focus Groups | Undertaking a 3-week RHM changed students’ perceptions of rural health and increased their knowledge of issues facing rural health practitioners  and patients. |
| Somers & Spencer 2012 | 81 | Australia | Impact of rural rotations on the choices of medical students in terms of rural career intentions. | 4-year Prospective controlled quasi-experiment | 58 graduates | Pre-and Post-survey.  Paper based questionnaire distributed during 2nd of a 5-year undergrad degree and repeated during the last week of the final year. | Graduate students who completed rural rotations had an increased likelihood to choose rural work upon graduation.  Rural rotations are an important factor; however, it is the nature of the students choosing to study in rural locations rather than experiences that is a stronger influence on rural work choices. |
| Matthews C et al. 2015 | 82 | New Zealand | Evaluation of a year 5 rural immersion program, looking at the early workforce outcomes of graduates who participated in the program. | Descriptive follow up study | 45 graduates | Post placement survey and follow up each year for four years. 2008-2011. | Follow up survey reported that 62% were working in rural or regional areas. 35.6% intended to return to Northland DHB (rural training area).  Graduates of the rural program are very likely to be working in rural and regional areas, and these graduates show an intention to work in general and rural medicine. |
| Myhre DL, Bajaj, S  2016 | 83 | Canada | The study looks at Longitudinal Integrated Clerkship (LIC). The study aims to identify the impact of a LIC on practice locations of graduates. | A prospective matched Cohort Study | A 3-year evaluation of a rural LIC involving the classes of 2009, 2010 and 2011. The study involved 34 students who completed a rural LIC. This group was compared against the control.  Study uses random selection of students to the LIC from applicant pool who self-select for the program. | Database was developed for both cohort groups. College information from websites from used to identify the graduates practice location.  Students were prospectively matched by background and sex, at the start of the clerkship between the cohort groups. | An association was identified between participation in a rural LIC and working in a rural location upon graduation. |
| Stagg et al. 2009 | 84 | Australia | Flinders University Parallel Rural Community Curriculum (PRCC) aimed at addressing the rural doctor workforce shortage. The PRCC program is a full academic year in rural general practices. The aim of the research was to ascertain the factors that influence the career choices. | Retrospective follow up study | Usable data were collected from 46 of the 86 contactable graduates (53%). | Alumni records of graduates were used, internet searches, telephone calls, rural doctor workforce agencies, medical registration and local contacts were all applied to ascertain the graduates working locations.  Of those identified graduates, a retrospective survey was provided. Surveys were completed by telephone interview or online completion. | The study reported that the PRCC is influencing graduates to choose a rural career path. |
| Eley & Baker 2007 | 85 | Australia | To study applied short term indicators (interest in a future rural career and internship choice) to assess how the University of Queensland rural clinical school is meeting its program aims. | Cross sectional study | 17/28 pre-surveys  27/28 post surveys  Students at the University of Queensland. | Pre-and post-Questionnaires | The study reported a positive effect of the program and an interest in pursuing a rural career. Study highlighted the need for long term tracking of the graduates. |
| Jamar E et al. 2014 | 86 | Australia | Tracks the early career movements of graduates of a rural training, to assess the impact on workforce choices. | Retrospective tracking study | 74 graduates  Grad survey of past students who completed a Rural Clinical Training Scheme Program – one year in a rural location in their 5^th^ year | Online questionnaire | Between 2009 and 2012 between 20.8% and 34.1% of respondents were located in a rural area.  More than 50% of respondents have worked in a rural area since graduation, and 85% reported increased rural intentions. |
| Eley D & Baker P 2006 | 87 | Australia | Study provides preliminary results from a longitudinal study which aims to identify the ways that Rural Clinical Schools can better prepare for rural employment. The study considered the impact of the rural clinical division on desire to pursue rural medicine | Descriptive study | 26 students completed their 4th year training at the RCD in  either the Hospital A (10 students) or Hospital B  (16 students). The sample size equals 25 because one  questionnaire from Hospital A was not completed. | Exit Questionnaire to evaluate perceptions of the students 4^th^ year in a rural location. Also, the study reported the graduate internship choices. | The study reported increased rural intentions post intervention. They reported that the rural training program was very popular with non-rural background students.  . |
| Lee YH et al. 2011 | 88 | Australia | The study reviewed the 4-year rural program at the Australian National University (ANU) to assess the impact of elective and compulsory program components on students’ rural intentions.  Variety of structured rural placements (8 to 4 weeks in duration). | Descriptive study | In total, 40 students from a cohort of 88 (45%) responded | Graduating cohort completed a voluntary online survey with 33 with forced answers | Reported increased rural intentions in rural placement participants.  The study supports the importance of compulsory rural health experiences in increasing students interest in rural health careers. In addition, they reported that all medical students should be targeted by a rural curriculum, not just those who are interested in rural work.  Only 15% of respondents chose a rural internship despite 80% indicating future rural employment. Therefore, no evidence in this study yet, that intentions translate into actual rural employment. |
| Birden HH & Wilson I 2012 | 89 | Australia | Study looks at a long term rural placement program at the University of Western Sydney (UWS), Included in the UWS curriculum is a longitudinal immersion experience in rural practice settings. This study looks at the first-year cohort of the UWS medical school long term rural placement students. | Descriptive study | 21 students who undertook a rural placement in their final year of the UWS medical program. | Post Questionnaire – 46 item quantitative survey with 37 closed-ended and nine open ended questions. Based on a validated survey. | The results indicate that the UWS Program increased the students’ receptiveness to a rural medical career. Study shows the program is off to a positive start. |
| Gerber & Landau 2010 | 90 | Australia | The Medical Schools Outcomes Database (MSOD) is a system that tracks all students from Australian and New Zealand medical schools through to their graduation and employment years. This study outlines the MSOD program and reports on the methodological factors, how it will inform an increased understanding of workforce changes and reports on the practice intentions from the 2005 pilot. | Descriptive study | Not specific | MSOD is four phases:  1)pre-questionnaire  2) clinical attachments, electives, and student activities are tracked via students  3) Exit questionnaire  4) Tracking through intern and vocational training years will occur in the future. | Rural placements led to associations with rural intentions. Future work will track the actual work outcomes of the graduates. |
| Williamson MI et al. 2012 | 91 | New Zealand | Study looking at the rural intentions and attitudes to rural health of graduates from a Rural Program. | Longitudinal tracking project  (cohort study) | 177 graduates  Two control groups of students from other schools. | Followed up tracking project using a mailed questionnaire to graduates years after qualifying.  Applied the data from a previous post survey. | 56% of respondents from Dunedin reported a positive influence compared with 24% from Christchurch and 15% Wellington.  Study concluded that the positive impact of a rural placement program persists into postgraduate years.  Further research is required to identify the specific features of the undergrad placement that positively impact on the students’ attitudes. |
| Denz-Penhey H et al. 2005 | 92 | Australia | Discusses multiple findings across 2 years, using three different cohorts. | Qualitative study  Comparing 3 study groups | (1) students based long term in one centre (with only a few days  away at a time);  (2) students based long term in one centre with short-term rotations of 3-6 weeks away from home base;  (3) week rotations without a home base. | Evaluation at the end of each 6-week placement.  Semi-structured interviews by phone  Questionnaires | Short rotations are less efficient than longer placements for increasing the rural workforce capacity.  The longer students live and work in a rural location, the more their emotional attachment builds up and connects them to the community. Students on short rotations do not make these local connections. |
| Deveney K et al.  2013 | 93 | United States | The study aimed to identify if 4th year residents trained in a rural setting, are more likely to enter general surgery practice in a rural setting, than those who were not trained in a rural setting.  Intervention involved a yearlong rural surgery training. | Descriptive study | 70 surgical residents in 2002.  11 rural Intervention.  59 (non-rural control).  2 groups- rural trained and other (not rural trained).  . | Age, sex, and initial practice plans, fellowship and practice characterises were the key outcome measures. | 91% (10/11) of the rural trained residents were more likely to enter rural practice than the non-rural trained cohort 47% (28/59).  The study also reported that the increase in likelihood for rural practice from the rural training, was independent of initial specialty plans. The rural resident training may assist in addressing the workforce gap in rural locations. |
| Greer, T et al. 2016 | 94 | United States | Workforce study addressing the needs of Washington, Wyoming, Alaska, Montana, and Idaho (WWAMI) region. A Targeted Rural Underserved Track (TRUST) 4-year curriculum Program was developed in 2008.  This study evaluates the TRUST program by looking at where the first graduates from it are working. | Longitudinal Tracking Project  The program provides a longitudinal continuity experience with students returning to a single rural site or small city. | From 2009, 123 students have been accepted into the program. In this period, 33 have graduated. | Study developed a tracking system to collect data on matriculation and then eventual working practice. | Of the 33 graduates, 30 (90.9%) entered needed regional specialties as defined by regional workforce needs  15 (50.0%) of these 30 graduates remained in the WWAMI region for their residency training.  Program is in its early stages and a robust evaluation program, additional funding, and developing linkages with regional and rural residency programs are planned. |
| Petrany SM &, Gress T 2013 | 95 | United States | Program set within the Marshall University Family Medicine Residency (MUFMR), which implemented its rural track (RT) in 1994, to address the mal-distribution of clinicians in rural areas and West Virginia.  The study looked at the impact of the RT on training outcomes, academics and curricula. | Retrospective Longitudinal Study | 174 MUFMR graduates who entered the residency program from 1994 to 2006. Included graduates 10 years prior (control) and the RT cohort who had graduated and been in practice for at least 1 year | Practice location was identified, and the academic performance of the cohorts was compared. | Of 174 MUFMR grads, 106 entered the residency program, with 12 completing the RT and 94 were the control group.  80% (10) of the RT graduates went onto practice in a rural location, compared with 40.4% (38) from the control group.  The RT provided an association with rural employment, and the graduates were more likely to work rurally than the control cohort. |
